# Real-world insights into young-onset gastroesophageal adenocarcinoma: an all-Ireland population-based cancer registry analysis

**DOI:** 10.1016/j.esmogo.2026.100325

**Published:** 2026-04-17

**Authors:** O. Deac, M.T. Redaniel, J. McDevitt, A. Brennan, D. Fitzpatrick, H.G. Coleman, R.C. Turkington, C. Donohoe, R. Narayanasamy, J.A. Elliott, J. Reynolds, J. O’Sullivan, M.A. Lowery

**Affiliations:** 1 Trinity St James’s Cancer Institute, St James’s Hospital, Dublin, Ireland; 2 School of Medicine, Trinity College Dublin, Dublin, Ireland; 3 National Cancer Registry Ireland, Cork, Ireland; 4 Northern Ireland Cancer Registry, Belfast, UK; 5 Centre for Public Health, School of Medicine, Dentistry and Biomedical Sciences, Queens University Belfast, Belfast, UK

**Keywords:** population-based cancer registry, incidence trends, socioeconomic deprivation, oesophageal adenocarcinoma, gastric adenocarcinoma, young-onset

## Abstract

**Background:**

Population-based studies report a rising incidence of young-onset (YO) oesophageal and gastric cancer. We examined clinicopathological features, treatment, and outcomes in YO (18-50 years), midlife-onset (MO, 50-70 years), and late-onset (LO, >70 years) oesophageal and gastric adenocarcinoma.

**Methods:**

Population-based data were collected from the National Cancer Registry Ireland (NCRI) and Northern Ireland Cancer Registry (NICR). Incidence trends were determined using Joinpoint regression. Kaplan–Meier curves, log-rank tests, and multivariable Cox regression were used to assess survival differences. Multivariable Cox regression was adjusted for key clinical covariates, and excluded patients with missing covariates.

**Results:**

We analysed 21 706 patients diagnosed with oesophageal or gastric adenocarcinoma from 1999 to 2022. YO gastric tumours were more diffuse (YO 26.6% versus MO 13.8% versus LO 10.4%, *P* < 0.001) and poorly differentiated (YO 60.6% versus MO 56.4% versus LO 55.0%, *P* < 0.0001); and presented more frequently with stage IV disease (YO 53.7% versus MO 46.4% versus LO 44.6%, *P* < 0.001). Incidence rates increased significantly across all age groups for lower oesophageal cancer: YO [average annual percentage change (AAPC) 2.6%, 95% confidence interval (CI) 1.1% to 4.3%], MO (AAPC 3.3%, 95% CI 2.4% to 4.4%), and LO (AAPC 2.1%, 95% CI 1.2% to 3.1%). Gastric cancer incidence was stable in YO but declined in the MO (AAPC −2.6%) and LO (AAPC −2.1%) groups. LO had poorer overall survival versus YO [hazard ratio 1.84, 95% CI 1.71% to 1.98%), *P* < 0.001, multivariable Cox analysis].

**Conclusion:**

Oesophageal adenocarcinoma incidence is rising across all age groups, while gastric adenocarcinoma incidence was stable in the YO population and declining in older adults. Despite more advanced and aggressive disease at presentation, YO patients had better overall survival.

## Highlights

• Lower oesophageal adenocarcinoma incidence increased across all ages (AAPC ∼2%-3%), including young-onset.
• Gastric adenocarcinoma incidence was stable in young-onset but declined in older groups.
• Oesophageal and gastric adenocarcinoma showed a strong deprivation gradient.
• Young-onset cases more often had stage IV disease and diffuse/poorly differentiated tumours.
• Young-onset patients had a modest survival advantage after adjustment.

## Graphical abstract

**Figure:**
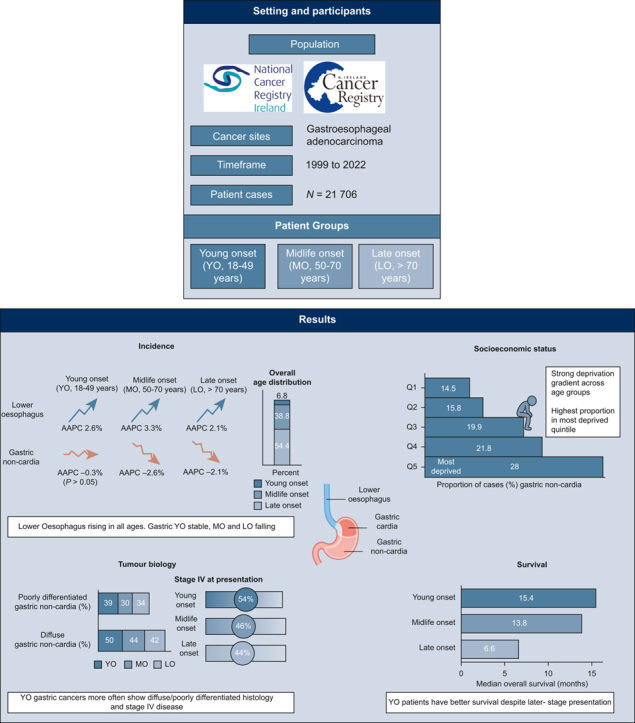

## Introduction

Oesophageal adenocarcinoma (OAC), oesophagogastric junction (OGJ), and gastric cancer (GC) represent a major global health burden, together accounting for >1 million cancer-related deaths worldwide in 2022.^1^ The number of cancer-related deaths are projected to increase by 61.8% in oesophageal cancer^2^ and 71% in GC.^3^

While the majority of oesophagogastric tumours are diagnosed in patients ≥60 years and older, several recent studies report a growing proportion of tumours being diagnosed in younger adults. Importantly, these trends vary by geography, anatomical subsite, and histology. US datasets have suggested increases in young-onset (YO) disease, including reports of rising GC incidence among women <50 years.^4^^,^^5^ In contrast, European evidence remains comparatively limited due to smaller case numbers and less granular subsite–histology classification in many datasets, although available European registry and multi-country data support heterogeneous age- and sex-specific patterns.^6^, ^7^, ^8^ These trends are of particular concern as YO (<50 years at diagnosis) patients often present with advanced disease,^4^^,^^9^^,^^10^ distinct clinicopathological features,^4^^,^^5^^,^^10^^,^^11^ and experience socioeconomic and health care-access disparities, including differences by insurance status and race/ethnicity.^12^^,^^13^

Diagnostic delay remains a major challenge in the recognition of YO cancers, particularly for malignancies traditionally associated with older age such as oesophageal and gastric adenocarcinoma. A meta-analysis by Castelo et al.^14^ found that time from symptom onset to diagnosis in YO colorectal cancer varied widely—from 50 to 450 days across 20 studies—illustrating prolonged pre-diagnostic intervals in younger adults.

Multiple risk factors contribute to OAC and GC, several of which appear to be more prevalent in younger patients. *Helicobacter pylori* infection is one of the main risk factors (75% attributable risk) for GC, playing a role in both intestinal and diffuse the gastric carcinogenesis.^15^ A large meta-analysis including YO GC cases reported *H. pylori* positivity in ∼60%,^16^ and several regional studies have observed higher infection rates among young females.^17^^,^^18^ This may contribute to the rising incidence of GC noted in younger women.

Smoking shows a dose-response increase in risk for both gastric (particularly cardia) and OAC.^19^ YO patients with lower-third OAC had higher rates of smoking, obesity, and drug use than older groups.^20^ Consistent with this, Zhang et al. identified smoking and high-salt diets as leading modifiable contributors to the YO GC burden globally.^21^ Additionally, Epstein–Barr virus (EBV)-positive gastric carcinoma accounts 8%-10% of GC,^22^ with emerging evidence suggesting that EBV-positive tumours occur more frequently in younger patients,^5^ implying possible age-specific immune or epigenetic susceptibilities. Together, these findings suggest that the increasing burden of YO gastroesophageal adenocarcinoma likely reflects both delayed recognition and a complex interplay of infections, lifestyle factors, and molecular susceptibilities unique to younger patients.

Treatment intensity and multimodal approaches are notably more prevalent among YO patients in both OAC and GC,^23^^,^^24^ however meta-analyses suggest no overall age advantage.^25^^,^^26^ We conducted a retrospective study with individual patient-level data from national cancer registries in the Republic of Ireland (ROI) and Northern Ireland (NI). We assessed socioeconomic status, incidence trends, stage at presentation, disease features, treatment patterns, and survival outcomes in upper gastrointestinal (UGI) cancer with a focus on YO patients (<50 years) with OAC or GC cancer subtypes, between 1999 and 2022.

## Methods

### Data sources and methods

This population-based study used pseudo-anonymized individual-level data from the National Cancer Registry Ireland (NCRI) and Northern Ireland Cancer Registry (NICR), both with statutory nationwide coverage. We identified all adults (≥18 years) diagnosed with gastro-oesophageal adenocarcinoma between 1 January 1999 and 31 December 2022 (censor date), defined by International Classification of Diseases (ICD)-10 codes C15.0-C15.9 (oesophagus) and C16.0-C16.9 (stomach), together with ICD-O-3 adenocarcinoma morphology codes: 8140-8149, 8190-8230, 8250-8384, 8440-8490, 8500-8552, and 8571-8576. Data were coded, standardized, and merged into a harmonized analytic dataset.

Socioeconomic status was assigned using each registry’s standard area-level index—NI Index of Multiple Deprivation and ROI Pobal HP Deprivation Index—and harmonized into common quintiles [from least (Q1) to most (Q5) socially deprived] for pooled analysis. Staging followed American Joint Committee on Cancer (AJCC)/Union for International Cancer Control (UICC) TNM (tumour–node–metastasis) editions: 4th (1999-2001), 5th (2002-2013), and 7th (2014-2022) for analysis. Stage was harmonised to grouped stage (I-IV) to enable comparability across editions.

### Statistical analysis

We described patients by sociodemographic and clinical characteristics as numbers and proportions. Differences in proportions were compared by chi-square tests and trends by Cochran–Armitage. A *P* value <0.05 was considered statistically significant.

Incidence was calculated as age-specific rates using mid-year population denominators for NI and ROI. Temporal trends were modelled using Joinpoint regression, allowing up to four joinpoints. The number and location of breakpoints were selected via the empirical-quantile permutation test with a weighted Bayesian information criterion. The annual percentage change (APC) and average annual percentage change (AAPC) with 95% confidence intervals (CIs) and *P* values were estimated using the program’s empirical quantile bootstrap (5001 resamples). Five-year survival was estimated from date of diagnosis to date of death or last follow-up. Kaplan–Meier curves, log-rank tests, and multivariable Cox regression were used to assess survival differences. Multivariable Cox regression was adjusted for key clinical covariates and analyses used complete cases, excluding patients with missing covariates (complete case analysis). Sensitivity analyses (Supplementary Table S2B, available at https://doi.org/10.1016/j.esmogo.2026.100325) included (i) alternative age specifications (<40 years/40-70 years/>70 years and age modelled continuously, reported per 10-year increase), and (ii) additional adjustment for registry-recorded treatment receipt (surgery, chemotherapy, radiotherapy). Cohort denominators and events are summarised in Supplementary Table S2A, available at https://doi.org/10.1016/j.esmogo.2026.100325), including 50-70 years as the reference group. To explore potential competing risk effects, we repeated the final multivariable Cox models with administrative censoring at 1 and 5 years and compared overall survival (OS) with cause-specific survival using a registry cause-of-death indicator (1 = alive or died of another cause/other cancer; 2 = died of the cancer under study). Net survival estimates from NCRI (2014-2018) were compared with observed OS at 1 and 5 years as an external benchmark. Analyses were done using R v4.4.2.

### Ethics

Ethical approval was granted by the Faculty of Health Sciences Research Ethics Committee at Trinity College Dublin.

## Results

### Patient characteristics

A total of 21 706 patients were included in this study; 33.1% were diagnosed with OAC and 66.9% with GC (Table 1). Of the study population, 6.8% were classified as YO, 38.8% as midlife-onset (MO), and 54.4% as late-onset (LO). In the overall cohort, 69.8% of patients were male. Sex distribution varied by age group and location; males predominated in proximal sites (lower OAC/OGJ and cardia), whereas females more frequently presented with distal non-cardia GC across the three age groups (*P* < 0.001, Supplementary Figure S1A-C, available at https://doi.org/10.1016/j.esmogo.2026.100325). Geographically, 68.3% of patients were diagnosed in ROI.

**Table 1:** Baseline clinicopathologic characteristics of the oesophagogastric adenocarcinoma cancer cohort

Variable	Overall	YO age <50 *n* (%)	MO age 50-70 *n* (%)	LO age >70 *n* (%)	*P* value
Number of patients	21 706	1486 (6.8)	8414 (38.8)	11 806 (54.4)	
Sex					*P* < 0.001
Male	15 164 (69.8)	1029 (69.2)	6422 (76.3)	7713 (65.3)	
Female	6542 (30.2)	457 (30.7)	1992 (23.7)	4093 (34.7)	
Location					*P* < 0.001
North	6890 (31.7)	390 (26.2)	2623 (31.2)	3877 (32.8)	
South	14 816 (68.3)	1096 (73.7)	5791 (68.8)	7929 (67.1)	
Socioeconomic status					*P* < 0.001
Q1 Least deprived	3413 (16.2)	209 (14.1)	1244 (14.8)	1960 (16.6)	
2nd Quintile	3618 (17.2)	283 (19.0)	1452 (17.3)	1883 (15.9)	
3rd Quintile	4204 (19.9)	290 (19.5)	1627 (19.3)	2287 (19.4)	
4th Quintile	4627 (21.9)	318 (21.4)	1838 (21.8)	2471 (20.9)	
Q5 Most deprived	5224 (24.8)	338 (22.7)	2010 (23.9)	2876 (24.4)	
UGI subsets					*P* < 0.001
Oesophageal upper/middle	672 (2.8)	42 (2.8)	250 (37.2)	380 (3.2)	
Lower oesophagus/OGJ	6572 (30.3)	452 (30.4)	3043 (46.3)	3077 (26.0)	
Gastric cardia	4840 (22.3)	324 (23.0)	1928 (39.8)	2588 (23.9)	
Gastric non-cardia	5775 (26.6)	407 (27.4)	1958 (33.9)	3410 (28.8)	
Gastric NOS	3878 (17.8)	261 (17.6)	1235 (32.1)	2351 (19.9)	
Morphology					*P* < 0.001
Intestinal	18 188 (83.8)	1049 (70.6)	6948 (82.6)	10 191 (86.3)	
Diffuse	2784 (12.8)	395 (26.6)	1162 (13.8)	1227 (10.4)	
Other/unknown	734 (3.4)	42 (2.8)	304 (3.6)	388 (3.3)	
Tumour grade					*P* < 0.001
Well/Mod differentiated	9511 (44.1)	583 (39.8)	3783 (43.6)	5145 (45.0)	
Poorly differentiated	12 069 (55.9)	895 (60.2)	4890 (56.4)	6284 (55.0)	
Stage at presentation					*P* < 0.001
Known stage	14 706 (68.0)	1171 (78.8)	6404 (76.1)	7189 (60.9)	
Stage I	2179 (14.8)	124 (10.6)	870 (13.6)	1185 (16.5)	
Stage II	2025 (13.7)	132 (11.3)	799 (12.5)	1094 (15.2)	
Stage III	3755 (25.4)	286 (24.4)	1764 (27.5)	1705 (23.7)	
Stage IV	6805 (46.1)	629 (53.7)	2971 (46.4)	3205 (44.6)	
Treatment					*P* < 0.001
Surgery	8223 (37.9)	678 (45.6)	3901 (46.4)	3644 (30.9)	
Chemotherapy	8449 (38.9)	948 (63.8)	4667 (55.5)	2834 (24.0)	
Radiotherapy	4156 (19.1)	361 (24.3)	1973 (23.4)	1822 (15.4)	

Data are presented as counts and percentages within each age group. For stage % are generated on the subset of patients with a known stage. Global *P* values compare the distribution across the three age bands—young-onset (YO, <50 years), midlife-onset (MO, 50-70 years) and late-onset (LO, >70 years)—using Pearson’s chi-square test. NOS, not otherwise specified; OGJ, oesophagogastric junction; UGI, upper gastrointestinal.

### Socioeconomic status

**Figure 2:**
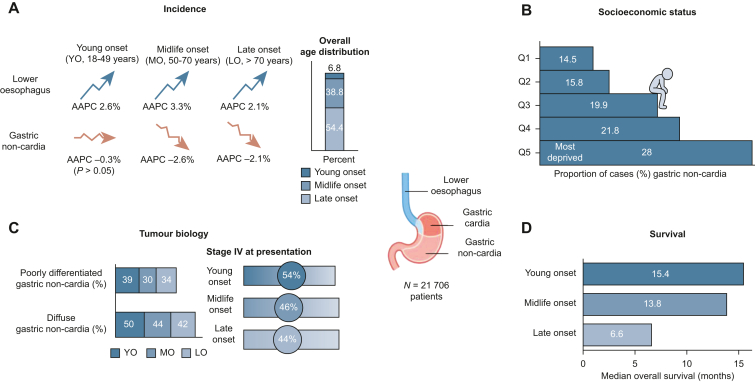
**Key findings in gastroesophageal adenocarcinoma by age at diagnosis.** (A) Incidence: YO lower oesophagus rising. Gastric YO stable, MO and LO falling. (B) Socioeconomic status: strong deprivation gradient in all ages. Highest rates in most deprived quintile and with more distal disease sites. (C) Tumour biology: YO with more distal disease present with more aggressive biology and advanced disease. (D) Survival: YO have better survival despite later stage presentation. AAPC, average annual percentage change; LO, late-onset; MO, midlife-onset; YO, young-onset;

Overall the highest case presentation was in the most socially deprived quintile with a stepwise increase across the five quintiles (Figure 2B, Supplementary Figure S2A, available at https://doi.org/10.1016/j.esmogo.2026.100325, chi-square tests, *P* < 0.01, Trend *P* < 0.001). This relationship was consistent across all primary cancer sites (Supplementary Figure S2B-F, available at https://doi.org/10.1016/j.esmogo.2026.100325) and age groups (Supplementary Figure S3A and B, available at https://doi.org/10.1016/j.esmogo.2026.100325).

### Clinicopathological features

**Figure 1:**
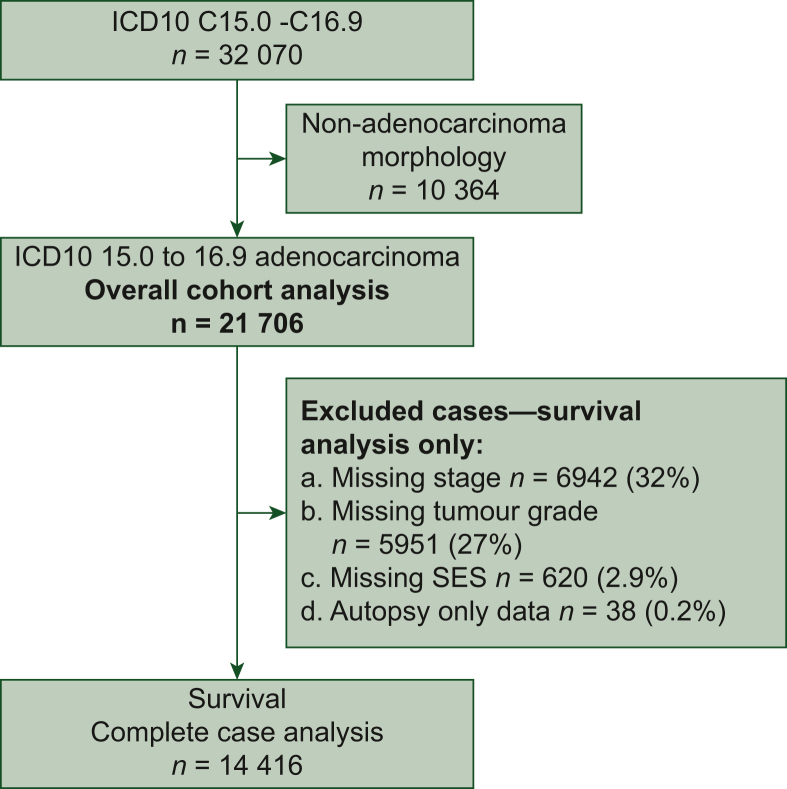
**Flow chart.** Flow diagram showing selection of the study cohort. From 32 070 oesophageal and gastric cancers (ICD-10 C15.0-C16.9), 21 706 adenocarcinomas were identified after exclusion of non-adenocarcinoma histology. Cases with missing tumour stage, tumour grade, socioeconomic status, or autopsy-only diagnosis were excluded, leaving 14 416 patients for the complete case survival analysis (numbers of excluded cases are not mutually exclusive). ICD, International Classification of Diseases; SES, socioeconomic status.

Across age groups morphological subtype varied (Table 1, *P* < 0.001; Figure 1C, Supplementary Figure S4A and B, available at https://doi.org/10.1016/j.esmogo.2026.100325). Intestinal-type adenocarcinoma had the highest frequency in all age groups (83.8%). The proportion of diffuse-type adenocarcinoma was higher in YO patients (26.6%) compared with MO (13.8%) or LO (10.4%) groups (Supplementary Figure S4A, available at https://doi.org/10.1016/j.esmogo.2026.100325, *P* < 0.001). Poorly differentiated cancers (grade 3-4) made up 55.9% of the total cohort, with the highest proportion seen in YO patients (Table 1, Supplementary Figure S4B, available at https://doi.org/10.1016/j.esmogo.2026.100325, *P* < 0.001). Overall the YO patients had the highest rate of metastatic disease at presentation (53.7%) versus 46.4% in the MO group and 44.6% in the LO group (Table 1, *P* < 0.0001). Stage IV cancers in the YO patients were more frequent in the gastric adenocarcinoma subgroups (Supplementary Figure S5B-E, available at https://doi.org/10.1016/j.esmogo.2026.100325, all subsite-specific, chi-square, *P* < 0.012). Across the three GC subtypes examined, the proportion presenting with stage IV disease decreased stepwise from YO to MO to LO patients (Cochran–Armitage trend tests *P* < 0.007; Supplementary Figure S4E, available at https://doi.org/10.1016/j.esmogo.2026.100325).

### Incidence trends

Across all age groups, the incidence rate of lower oesophagus/OGJ adenocarcinoma rose, with AAPCs of 2.6% (YO), 3.3% (MO), and 2.1% (LO) (Figure 1A, Supplementary Figure S6A-E, available at https://doi.org/10.1016/j.esmogo.2026.100325 all *P* < 0.001), with the steepest increase seen in males. For gastric cardia tumours, the overall incidence rate was stable, with the exception of a decline in older men (APC −2.0%, *P* = 0.030). Gastric non-cardia YO incidence remained stable while MO and LO rates fell by AAPCs of −2.8% to −2.1% (*P* ≤ 0.017) across both sexes.

### Survival analysis

**Figure 3:**
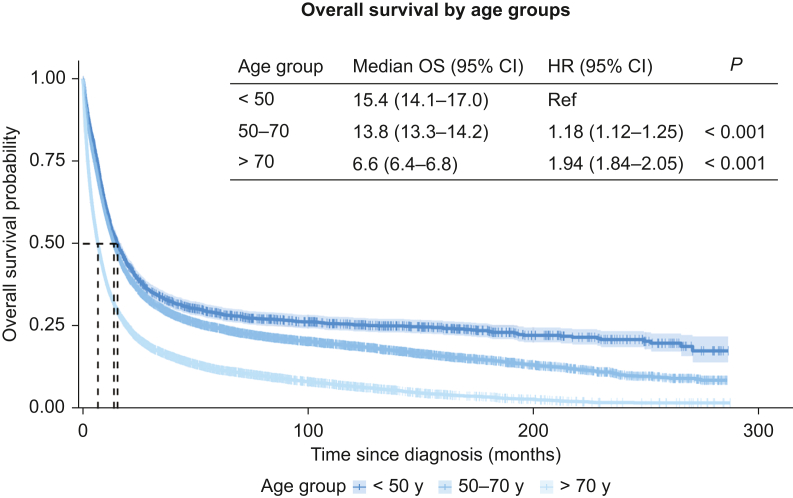
**Overall survival by age group.** Kaplan–Meier curves of overall survival (months since diagnosis) by age band with 95% CI. The inset table lists median OS (95% CI), age-group HRs (95% CI) from a univariate Cox model, and log-rank *P* values. CI, confidence interval; HR, hazard ratio; OS, overall survival.

Median OS differed significantly by age; 15.4 months (95% CI 14.1-17.0 months) for YO, 13.8 months (95% CI 13.3-14.2 months) for MO, and 6.6 months (95% CI 6.4-6.8 months) for LO cancers (Figure 3). Median OS also differed by tumour subsite and stage, but not by sex or socioeconomic status (Supplementary Table S1, available at https://doi.org/10.1016/j.esmogo.2026.100325). After adjusting for demographics, tumour characteristics and anatomical subsite, YO patients had significantly better OS than the >70 years subgroup (Table 2, *P* < 0.001). Cohort denominators and events for the complete case Cox analysis are summarised in Supplementary Table S2A, available at https://doi.org/10.1016/j.esmogo.2026.100325. Sensitivity analyses showed consistent findings using alternative age definitions (<40 years, 40-70 years, >70 years) and when modelling age continuously (per 10-year increase using age-band midpoints). Additional adjustment for treatment receipt (surgery, chemotherapy, radiotherapy) attenuated the age-associated hazard ratios but did not change the overall interpretation (Supplementary Table S2B, available at https://doi.org/10.1016/j.esmogo.2026.100325, Sensitivity 3). Treatment receipt also varied by age and subsite, with lower uptake of surgery, chemotherapy, and radiotherapy in older patients (Supplementary Figure S7, available at https://doi.org/10.1016/j.esmogo.2026.100325). OS and cancer-specific models at 1 and 5 years showed similar age effects at 1 year with attenuation at 5 years, consistent with increasing competing mortality over time (Supplementary Table S3, available at https://doi.org/10.1016/j.esmogo.2026.100325). In external benchmarking, NCRI net survival estimates (2014-2018) were broadly similar to observed OS at 1 year, with greater divergence at 5 years, particularly in older age bands, consistent with increasing competing mortality over time (Supplementary Table S4, available at https://doi.org/10.1016/j.esmogo.2026.100325).

**Table 2:** Multivariable Cox proportional hazards

Co-variable	HR (95% CI)	*P* value
Age group (years)		
<50	Ref	
50-70	1.13 (1.05-1.21)	<0.001
>70	1.84 (1.71-1.98)	<0.001
Sex		
Female	Ref	
Male	0.96 (0.92-1.00)	0.061
Location		
North	Ref	
South	0.99 (0.94-1.04)	0.614
SES		
Quintile 1	Ref	
Quintile 2	1.02 (0.95-1.09)	0.581
Quintile 3	1.05 (0.98-1.11)	0.150
Quintile 4	1.05 (0.99-1.12)	0.096
Quintile 5	1.11 (1.04-1.17)	<0.001
Unknown	1.00 (0.89-1.12)	0.950
Morphology		
Diffuse	Ref	
Intestinal	0.93 (0.88-0.99)	0.013
Mucinous	1.01 (0.89-1.15)	0.889
Other/unknown	0.91 (0.78-1.05)	0.196
Stage		
Stage I	Ref	
Stage II	1.72 (1.58-1.86)	<0.001
Stage III	2.74 (2.55-2.95)	<0.001
Stage IV	7.49 (6.99-8.03)	<0.001
Tumour grade		
Grade 1	Ref	
Grade 2	1.12 (0.99-1.28)	0.080
Grade 3	1.37 (1.20-1.56)	<0.001
Grade 4	1.20 (0.88-1.63)	0.257
Unknown	1.36 (1.19-1.56)	<0.001
UGI subset		
Oesophagus upper middle	Ref	
Oesophagus lower third/OGJ	0.81 (0.73-0.91)	<0.001
Gastric cardia	0.74 (0.66-0.83)	<0.001
Gastric non cardia	0.74 (0.66-0.83)	<0.001
Gastric NOS	0.87 (0.78-0.98)	0.023
Year (continuous)	0.98 (0.97-0.98)	<0.001

Model for overall survival Cox proportional hazards model adjusting for all listed covariates. Case complete for stage only *n* = 14 416. HRs <1 indicate reduced risk of death compared with the reference category. Continuous ‘Year’ reflects the calendar year of diagnosis (per year change). CI, confidence interval; HR, hazard ratio; NOS, not otherwise specified; OGJ, oesophagogastric junction; ref, reference, SES, socioeconomic status; UGI, upper gastrointestinal.

The key results in this study are summarized in Figure 2A-D.

## Discussion

In this study we investigated the changing epidemiological and clinical characteristics of OC and GC in Ireland over the last two decades. YO gastric and OAC cases accounted for 6.8% of our dataset; this is consistent with prior publications reporting proportions of between 5% and 30%.^4^^,^^5^^,^^9^^,^^13^ Among female patients, distal gastric tumours [non-cardia or not otherwise specified (NOS)] represented 70.3% of YO cancers, compared with 56.2% and 57.5% of tumours in midlife and older females, respectively. This sex- and age-specific subsite pattern parallels the observations of Niu et al.,^26^ who reported a disproportionate burden of distal GC in young women.

Young patients in our study were more likely to have diffuse/signet-ring and poorly differentiated gastric tumours especially in non-cardia and NOS sites, consistent with prior studies which identified more aggressive histology subtypes in YO patients.^5^^,^^9^^,^^10^^,^^13^^,^^26^ More than half of the YO group presented with metastatic (stage IV) disease, significantly more than average-onset (AO) and LO groups, as previously reported.^4^^,^^9^^,^^27^^,^^28^ Potential contributing factors to this include, diagnostic delay in younger adults (with reflux, dysphagia, or dyspepsia being attributed to benign conditions) and a more aggressive phenotype with early metastatic spread, as well as factors linked to deprivation and access. These explanations should be interpreted as hypotheses rather than inferences from the present data, as symptom and diagnostic interval information was not available in our registry dataset.

We demonstrate a clear deprivation gradient, with case proportions rising stepwise across increasing deprivation levels overall, by age, and by cancer site. This aligns with international evidence identifying socioeconomic deprivation as a consistent risk factor for GC^29^ and European data documenting cancer incidence inequalities.^30^ While YO-specific socioeconomic status data in Western Europe remain limited, US data show YO GC disproportionately affects Hispanic and black populations with differences in insurance and treatment.^12^^,^^31^, ^32^, ^33^ The gradient observed here is consistent with both NCRI and NICR reports: in ROI, stomach cancer incidence was 48% higher in men and 63% higher in women in the most versus least deprived areas,^34^ while in Northern Ireland, oesophageal and stomach cancer incidence were 17.7% and 35.8% above average in the most deprived quintile.^35^^,^^36^ Our all-island, age-stratified analysis provides robust patient-level evidence that social determinants influence YO oesophagogastric cancer across both health systems.

Incidence trends in our cohort reproduce the various trajectories observed in prior studies across different geographic locations. In cancers of the lower oesophagus/OGJ, rates are rising across all ages, steepest in AO males and also evident in YO males, in line with observed increases in oesophageal/OGJ adenocarcinoma^37^^,^^38^ and YO-specific increases around 2%-3% annually.^4^^,^^39^ In contrast, GC shows marked declines in AO/LO with stable or increasing YO rates,^40^ consistent with global reductions attributed to sanitation, *H. pylori* detection/eradication, and dietary shifts,^38^^,^^41^ alongside heterogeneous YO patterns by region and subsite.^11^^,^^42^ The practical implication is that the relative contribution of YO disease to overall GC burden is increasing, even where absolute YO rates are stable.

OS was longest in the YO population, and this difference persisted after adjustment for stage, subsite, and morphology. There is conflicting evidence in the literature regarding survival outcomes in YO patients. Some OAC analyses reported worse survival outcomes in YO patients,^4^ whereas others reported equal or better adjusted survival in younger patients.^27^^,^^43^ One meta-analysis reported that in OAC, pooled 5-year survival and mortality risk were comparable by age.^25^ A separate meta-analysis in GC likewise showed no overall difference in pooled OS by age, with any survival advantage for younger patients confined to those treated with gastrectomy.^26^ Our all-island data suggest a modest OS difference favouring younger patients, but this should be interpreted cautiously given competing mortality and treatment selection. Registry data capture treatment receipt only (not intensity/sequence/intent or comorbidity), and treatment-adjusted sensitivity analyses attenuated the age effect. Therefore, we interpret this as an OS difference by age rather than evidence of a cancer-specific survival advantage. This is supported by our OS versus cancer-specific and net survival comparisons, which show closer alignment at 1 year and greater divergence at 5 years, particularly in older age bands.

Key limitations of our study include missing data particularly for stage and grade, more common in earlier calendar years and among patients ≥70 years, with relatively little missingness in those <50 years. Survival for patients with missing versus known stage/grade was broadly similar (Kaplan–Meier curves overlapping, with medians only ∼2-4 months shorter in the missing group), and age group hazard ratios were very similar in sensitivity analyses using ‘unknown’ categories and stage-stratified models. Together, these findings suggest that while some bias is possible, missing data are unlikely to change the age-related incidence and survival patterns observed, although residual confounding (e.g. comorbidity, performance status symptom burden, diagnostic intervals), coding changes, and treatment selection remain potential sources of bias. Changes in staging and management over time (including TNM edition updates, evolving diagnostic/staging pathways, and changes in multimodality treatment standards) may also limit comparability across studies.

Our findings provide a robust, population-based characterization of YO gastroesophageal adenocarcinoma. The observed epidemiologic patterns, rising incidence of oesophageal and OGJ adenocarcinoma, relatively stable YO gastric rates, and a persistent socioeconomic gradient, mirror international trends and highlight the growing clinical importance of this subgroup. The modest survival advantage seen in younger patients, despite later stage at diagnosis, likely reflects treatment intensity and baseline fitness. Collectively, these results underscore the need for strategies that promote earlier recognition and timely diagnosis in younger adults presenting with upper gastrointestinal symptoms. This includes raising public and clinician awareness through dedicated patient and public involvement initiatives aimed at improving symptom appraisal and health care engagement, with the ultimate goal of increasing presentation at earlier curable stages.

## References

[1] Bray F., Laversanne M., Sung H. (2024). Global cancer statistics 2022: GLOBOCAN estimates of incidence and mortality worldwide for 36 cancers in 185 countries. CA Cancer J Clin.

[2] Morgan E., Soerjomataram I., Rumgay H. (2022). The global landscape of esophageal squamous cell carcinoma and esophageal adenocarcinoma incidence and mortality in 2020 and projections to 2040: new estimates from GLOBOCAN 2020. Gastroenterology.

[3] Morgan E., Arnold M., Camargo M.C. (2022). The current and future incidence and mortality of gastric cancer in 185 countries, 2020–40: a population-based modelling study. eClinicalMedicine.

[4] Codipilly D.C., Sawas T., Dhaliwal L. (2021). Epidemiology and outcomes of young-onset esophageal adenocarcinoma: an analysis from a population-based database. Cancer Epidemiol Biomarkers Prev.

[5] Bergquist J.R., Leiting J.L., Habermann E.B. (2019). Early-onset gastric cancer is a distinct disease with worrisome trends and oncogenic features. Surgery.

[6] Radkiewicz C., Asplund J., Lagergren J. (2023). Incidence trends and survival in early-onset esophagogastric adenocarcinoma: a Swedish population-based cohort study. Cancer Epidemiol Biomarkers Prev.

[7] Al-Kaabi A., Baranov N.S., van der Post R.S. (2022). Age-specific incidence, treatment, and survival trends in esophageal cancer: a Dutch population-based cohort study. Acta Oncol.

[8] Ben-Aharon I., Fokter Dovnik N., van Laarhoven H.W.M. (2025). Sex differences in the incidence trends of early-onset gastrointestinal cancer—the European/Mediterranean perspective. ESMO Gastrointest Oncol.

[9] Cheng L., Chen S., Wu W. (2020). Gastric cancer in young patients: a separate entity with aggressive features and poor prognosis. J Cancer Res Clin Oncol.

[10] Tavares A., Gandra A., Viveiros F., Cidade C., Maciel J. (2013). Analysis of clinicopathologic characteristics and prognosis of gastric cancer in young and older patients. Pathol Oncol Res.

[11] Tan N., Wu H., Cao M. (2024). Global, regional, and national burden of early-onset gastric cancer. Cancer Biol Med.

[12] Holowatyj A.N., Ulrich C.M., Lewis M.A. (2019). Racial/ethnic patterns of young-onset noncardia gastric cancer. Cancer Prev Res (Phila).

[13] Boys J.A., Oh D.S., Lewis J.S., DeMeester S.R., Hagen J.A. (2015). Esophageal adenocarcinoma in patients younger than 40 years: a two-decade experience at a public and private hospital. Am Surg.

[14] Castelo M., Sue-Chue-Lam C., Paszat L. (2022). Time to diagnosis and treatment in younger adults with colorectal cancer: a systematic review. PLoS One.

[15] Parsonnet J., Friedman G.D., Vandersteen D.P. (1991). Helicobacter pylori infection and the risk of gastric carcinoma. N Engl J Med.

[16] Li Y., Hahn A.I., Laszkowska M., Jiang F., Zauber A.G., Leung W.K. (2024). Clinicopathological characteristics and risk factors of young-onset gastric carcinoma: a systematic review and meta-analysis. Clin Transl Gastroenterol.

[17] Naser N.K.A.A., Bashir M.B.M., Ali A.S.M.A. (2025). Prevalence and associated risk factors of Helicobacter pylori infection among medical students at Shendi university, Sudan. BMC Gastroenterol.

[18] Alshareef S.A., Hassan A.A., Abdelrahman D.N., AlEed A., Al-Nafeesah A., Adam I. (2023). The prevalence and associated factors of Helicobacter pylori infection among asymptomatic adolescent schoolchildren in Sudan: a cross-sectional study. BMC Pediatr.

[19] Rota M., Possenti I., Valsassina V. (2024). Dose-response association between cigarette smoking and gastric cancer risk: a systematic review and meta-analysis. Gastric Cancer.

[20] Rabeeah S., Mahdi A., Kumar V. (2025). Characteristics of early- versus late-onset esophageal adenocarcinoma: insights from the National Inpatient Sample 2016-2020. Ann Gastroenterol.

[21] Zhang X., Gao B., Wang W. (2025). Early-onset gastric cancer global burden profile, trends, and contributors. Cancer Biol Med.

[22] Cancer Genome Atlas Research Network (2014). Comprehensive molecular characterization of gastric adenocarcinoma. Nature.

[23] Song E.Y., Naffouje S.A., Saeed S. (2021). Esophageal cancer in young patients: does age affect treatment course and outcomes?. Ann Esophagus.

[24] Jiang Y., Xie J., Huang W. (2020). Chemotherapy use and survival among young and middle-aged patients with gastric cancer. Clin Transl Gastroenterol.

[25] Russell A., Mitchell S., Turkington R.C., Coleman H.G. (2024). Survival outcomes in early-onset oesophageal adenocarcinoma patients: a systematic review and meta-analyses. World J Gastroenterol.

[26] Niu P., Zhao L., Ling R., Zhao D., Chen Y. (2020). Clinicopathological characteristics and survival outcomes of younger patients with gastric cancer: a systematic review and meta-analysis. Transl Cancer Res.

[27] Lai H., Zheng J., Zhou G., Li Y. (2023). Clinical characteristics and prognostic outcomes for adenocarcinoma of esophagogastric junction in early-onset patients: a population-based appraisal. J Cancer Res Clin Oncol.

[28] Hsieh F.J., Wang Y.C., Hsu J.T., Liu K.H., Yeh C.N. (2012). Clinicopathological features and prognostic factors of gastric cancer patients aged 40 years or younger. J Surg Oncol.

[29] Rawla P., Barsouk A. (2019). Epidemiology of gastric cancer: global trends, risk factors and prevention. Prz Gastroenterol.

[30] Mihor A., Tomsic S., Zagar T., Lokar K., Zadnik V. (2020). Socioeconomic inequalities in cancer incidence in Europe: a comprehensive review of population-based epidemiological studies. Radiol Oncol.

[31] Torrejon N.V., Deshpande S., Wei W., Tullio K., Kamath S.D. (2022). Proportion of early-onset gastric and esophagus cancers has changed over time with disproportionate impact on black and Hispanic patients. JCO Oncol Pract.

[32] Yasinzai A.Q.K., Saeed A. (2025). Age-related differences in gastric adenocarcinoma from 2000-2020: a SEER database analysis. J Gastrointest Cancer.

[33] Sawas T., Manrique G.C., Iyer P.G., Wang K.K., Katzka D.A. (2019). Young adults with esophageal adenocarcinoma present with more advanced stage tumors and have shorter survival times. Clin Gastroenterol Hepatol.

[34] Bambury N., Brennan A., McDevitt J., Walsh P.M. (2023). https://www.ncri.ie/en/reports-publications/reports/cancer-inequalities-in-ireland-by-deprivation-2004-2018.

[35] Northern Ireland Cancer Registry (2024).

[36] Northern Ireland Cancer Registry (2024).

[37] Arnold M., Ferlay J., van Berge Henegouwen M.I., Soerjomataram I. (2020). Global burden of oesophageal and gastric cancer by histology and subsite in 2018. Gut.

[38] Giusti F., Martos C., Bettio M. (2024). Geographical and temporal differences in gastric and oesophageal cancer registration by subsite and morphology in Europe. Front Oncol.

[39] Schell D., Ullah S., Brooke-Smith M.E. (2022). Gastrointestinal adenocarcinoma incidence and survival trends in South Australia, 1990-2017. Cancers (Basel).

[40] Arnold M., Park J.Y., Camargo M.C., Lunet N., Forman D., Soerjomataram I. (2020). Is gastric cancer becoming a rare disease? A global assessment of predicted incidence trends to 2035. Gut.

[41] Wong M.C.S., Huang J., Chan P.S.F. (2021). Global incidence and mortality of gastric cancer, 1980-2018. JAMA Netw Open.

[42] Li Y., Hahn A.I., Laszkowska M., Jiang F., Zauber A.G., Leung W.K. (2024). Global burden of young-onset gastric cancer: a systematic trend analysis of the global burden of disease study 2019. Gastric Cancer.

[43] Rompen I.F., Nienhuser H., Crnovrsanin N. (2023). Clinical characteristics and oncological outcomes of surgically treated early-onset gastric adenocarcinoma – a retrospective cohort study. J Cancer.

