# Supplementary material for: Real-world insights into young-onset gastroesophageal adenocarcinoma: an all-Ireland population-based cancer registry analysis
Source: ESMO Gastrointest Oncol. 2026 Apr 17;12:100325. doi: 10.1016/j.esmogo.2026.100325 (PMC13098464; doi:10.1016/j.esmogo.2026.100325)
Supplement: Supplementary Tables [file mmc1.docx]

| **Age group (years)** | **Number of UGI cancer cases** | **Person years of follow-up** | **Number of deaths** | **Overall survival** | | |
| --- | --- | --- | --- | --- | --- | --- |
|  |  |  |  | **Unadjusted**  **HR (95 % CI)** | **Adjusted Model 1**  **HR (95 % CI)** | **Adjusted Model 2**  **HR (95 % CI)** |
| **Main analysis** |  |  |  |  |  |  |
| **<50** | **1,146** | **3,468** | **906** | **Ref** | **Ref** | **Ref** |
| **50– 70** | **6,256** | **18,271** | **4,879** | **1.18 (1.12–1.25)** | **1.13 (1.05–1.21)** | **1.06 (0.99–1.14)** |
| **>70** | **7,014** | **13,339** | **6,071** | **1.94 (1.84–2.05)** | **1.84 (1.71–1.98)** | **1.40 (1.30–1.50)** |
| ***Sensitivity analysis*** |  |  |  |  |  |  |
| **Middle-aged as reference category** |  |  |  |  |  |  |
| **<50** | **1,146** | **3,468** | **906** | **1.02 (0.95–1.09)** | **0.89 (0.82–0.95)** | **0.94 (0.88–1.01)** |
| **50–70** | **6,256** | **18,271** | **4,879** | **Ref** | **Ref** | **Ref** |
| **> 70** | **7,014** | **13,339** | **6,071** | **1.42 (1.36–1.47)** | **1.63 (1.57–1.69)** | **1.31 (1.26–1.37)** |
| **Categorising young-onset as <40** |  |  |  |  |  |  |
| **<40** | **275** | **851** | **217** | **Ref** | **Ref** | **Ref** |
| **40–70** | **7,127** | **20,888** | **5,568** | **0.95 (0.83–1.09)** | **1.30 (1.13–1.49)** | **1.08 (0.94–1.23)** |
| **>70** | **7,014** | **13,338** | **6,071** | **1.35 (1.18–1.55)** | **2.22 (1.93–2.55)** | **1.42 (1.24–1.63)** |

**Table S2A Multivariable Cox Proportional-Hazards for Overall survival in gastroesophageal cancer cases with complete information for stage (n=14,416)**

Model 1 Adjustments: sex (male/female), location (Northern Ireland / Republic of Ireland), Socio-economic status (area deprivation quintiles), morphology (diffuse/intestinal/mucinous/other or unknown), tumour grade (1, 2, 3, 4, unknown), tumour location (upper middle oesophagus / lower third oesophagus or oesophagogastric junction / gastric cardia / gastric non-cardia / gastric not otherwise specified), calendar year of diagnosis (continuous).

Model 2 Adjustments: Model 1 adjustments plus treatment: surgery (yes / no or unknown), radiotherapy (yes / no or unknown), chemotherapy (yes / no or unknown).

UGI= Upper Gastrointestinal; NOS = not otherwise specified; Ref = reference.

| **Co-Variable** | **Original** | **p Value** | **Co-Variable** | **Sensitivity 1** | **p Value** | **Co-Variable** | **Sensitivity 2** | **p Value** | **Co-Variable** | **Sensitivity 3** | **p Value** |
| --- | --- | --- | --- | --- | --- | --- | --- | --- | --- | --- | --- |
|  |  |  |  | **<40, 40-70, >70** |  |  | **Age as a continuous variable** | |  | **including Treatment as covariate** |  |
|  | HR (95 % CI) |  |  | HR (95 % CI) |  |  | HR (95 % CI) |  |  | HR (95 % CI) |  |
| **Age group** |  |  | **Age group** |  |  | **Age group** |  |  | **Age group** |  |  |
| <50 | Ref |  | <40 | Ref |  | Continuous | 1.29 (1.27–1.31) | <0.001 | <50 | Ref |  |
| 50–70 | 1.13 (1.05–1.21) | <0.001 | 40–70 | 1.30 (1.13–1.49) | <0.001 |  |  |  | 50–70 | 1.06 (0.99–1.14) | 0.094 |
| > 70 | 1.84 (1.71–1.98) | <0.001 | > 70 | 2.22 (1.93–2.55) | <0.001 |  |  |  | > 70 | 1.40 (1.30–1.50) | <0.001 |
| **Sex** |  |  | **Sex** |  |  | **Sex** |  |  | **Sex** |  |  |
| Female | Ref |  | Female | Ref |  | Female | Ref |  | Female | Ref |  |
| Male | 0.96 (0.92–1.00) | 0.061 | Male | 0.97 (0.93–1.01) | 0.121 | Male | 0.96 (0.92–1.00) | 0.055 | Male | 1.00 (0.96–1.05) | 0.878 |
| **Location** |  |  | **Location** |  |  | **Location** |  |  | **Location** |  |  |
| North | Ref |  | North | Ref |  | North | Ref |  | North | Ref |  |
| South | 0.99 (0.94–1.04) | 0.614 | South | 0.98 (0.94–1.03) | 0.488 | South | 0.99 (0.95–1.04) | 0.756 | South | 1.05 (1.00–1.10) | 0.047 |
| **SES** |  |  | **SES** |  |  | **SES** |  |  | **SES** |  |  |
| Quintile 1 | Ref |  | Quintile 1 | Ref |  | Quintile 1 | Ref |  | Quintile 1 | Ref |  |
| Quintile 2 | 1.02 (0.95–1.09) | 0.581 | Quintile 2 | 1.02 (0.96–1.09) | 0.519 | Quintile 2 | 1.03 (0.96–1.09) | 0.446 | Quintile 2 | 1.02 (0.96–1.09) | 0.529 |
| Quintile 3 | 1.05 (0.98–1.11) | 0.150 | Quintile 3 | 1.04 (0.98–1.10) | 0.235 | Quintile 3 | 1.04 (0.98–1.11) | 0.179 | Quintile 3 | 1.04 (0.98–1.10) | 0.221 |
| Quintile 4 | 1.05 (0.99–1.12) | 0.096 | Quintile 4 | 1.06 (1.00–1.12) | 0.062 | Quintile 4 | 1.06 (0.99–1.12) | 0.077 | Quintile 4 | 1.06 (1.00–1.13) | 0.056 |
| Quintile 5 | 1.11 (1.04–1.17) | <0.001 | Quintile 5 | 1.12 (1.06–1.19) | <0.001 | Quintile 5 | 1.12 (1.05–1.18) | <0.001 | Quintile 5 | 1.07 (1.01–1.14) | 0.020 |
| Unknown | 1.00 (0.89–1.12) | 0.950 | Unknown | SESUnknown | 1.01 (0.90–1.13) | Unknown | 1.01 (0.90–1.14) | 0.830 | Unknown | 0.95 (0.85–1.07) | 0.434 |
| **Morphology** |  |  | **Morphology** |  |  | **Morphology** |  |  | **Morphology** |  |  |
| Diffuse | Ref |  | Diffuse | Ref |  | Diffuse | Ref |  | Diffuse | Ref |  |
| Intestinal | 0.93 (0.88–0.99) | 0.013 | Intestinal | 0.95 (0.90–1.00) | 0.063 | Intestinal | 0.90 (0.85–0.95) | <0.001 | Intestinal | 0.90 (0.86–0.96) | <0.001 |
| Mucinous | 1.01 (0.89–1.15) | 0.889 | Mucinous | 1.02 (0.90–1.16) | 0.753 | Mucinous | 0.96 (0.85–1.10) | 0.576 | Mucinous | 1.05 (0.92–1.19) | 0.482 |
| Other/  Unknown | 0.91 (0.78–1.05) | 0.196 | Other/  Unknown | 0.97 (0.83–1.13) | 0.680 | Other/  Unknown | 0.89 (0.77–1.03) | 0.129 | Other/  Unknown | 0.87 (0.75–1.01) | 0.074 |
| **Stage** |  |  | **Stage** |  |  | **Stage** |  |  | **Stage** |  |  |
| Stage I | Ref |  | Stage I | Ref |  | Stage I | Ref |  | Stage I | Ref |  |
| Stage II | 1.72 (1.58–1.86) | <0.001 | Stage II | 1.72 (1.59–1.86) | <0.001 | Stage II | 1.72 (1.59–1.87) | <0.001 | Stage II | 2.02 (1.86–2.20) | <0.001 |
| Stage III | 2.74 (2.55–2.95) | <0.001 | Stage III | 2.73 (2.53–2.93) | <0.001 | Stage III | 2.75 (2.55–2.95) | <0.001 | Stage III | 3.56 (3.30–3.84) | <0.001 |
| Stage IV | 7.49 (6.99–8.03) | <0.001 | Stage IV | 7.42 (6.92–7.95) | <0.001 | Stage IV | 7.58 (7.07–8.13) | <0.001 | Stage IV | 7.76 (7.19–8.37) | <0.001 |
| **Tumour grade** |  |  | **Tumour grade** |  |  | **Tumour grade** |  |  | **Tumour grade** |  |  |
| Grade 1 | Ref |  | Grade 1 | Ref |  | Grade 1 | Ref |  | Grade 1 | Ref |  |
| Grade 2 | 1.12 (0.99–1.28) | 0.080 | Grade 2 | 1.14 (1.00–1.29) | 0.052 | Grade 2 | 1.14 (1.00–1.30) | 0.046 | Grade 2 | 1.13 (0.99–1.28) | 0.075 |
| Grade 3 | 1.37 (1.20–1.56) | <0.001 | Grade 3 | 1.37 (1.20–1.55) | <0.001 | Grade 3 | 1.39 (1.22–1.58) | <0.001 | Grade 3 | 1.37 (1.21–1.56) | <0.001 |
| Grade 4 | 1.20 (0.88–1.63) | 0.257 | Grade 4 | 1.15 (0.84–1.57) | 0.391 | Grade 4 | 1.23 (0.90–1.68) | 0.191 | Grade 4 | 1.35 (0.99–1.85) | 0.057 |
| Unknown | 1.36 (1.19–1.56) | <0.001 | Unknown | 1.35 (1.18–1.54) | <0.001 | Unknown | 1.37 (1.20–1.57) | <0.001 | Unknown | 1.19 (1.04–1.36) | 0.011 |
| **UGI subset** |  |  | **UGI subset** |  |  | **UGI subset** |  |  | **UGI subset** |  |  |
| Oesophagus Upper Middle | Ref |  | Oesophagus Upper Middle | Ref |  | Oesophagus Upper Middle | Ref |  | Oesophagus Upper Middle | Ref |  |
| Oesophagus Lower Third/OGJ | 0.81 (0.73–0.91) | <0.001 | Oesophagus Lower Third/OGJ | 0.81 (0.72–0.90) | <0.001 | Oesophagus Lower Third/OGJ | 0.81 (0.73–0.91) | <0.001 | Oesophagus Lower Third/OGJ | 0.88 (0.79–0.99) | 0.029 |
| Gastric Cardia | 0.74 (0.66–0.83) | <0.001 | Gastric Cardia | 0.75 (0.67–0.84) | <0.001 | Gastric Cardia | 0.74 (0.67–0.83) | <0.001 | Gastric Cardia | 0.86 (0.77–0.97) | 0.011 |
| Gastric Non Cardia | 0.74 (0.66–0.83) | <0.001 | Gastric Non Cardia | 0.74 (0.66–0.83) | <0.001 | Gastric Non Cardia | 0.74 (0.66–0.82) | <0.001 | Gastric Non Cardia | 0.87 (0.78–0.98) | 0.019 |
| Gastric NOS | 0.87 (0.78–0.98) | 0.023 | Gastric NOS | 0.88 (0.79–0.99) | 0.038 | Gastric NOS | 0.87 (0.77–0.98) | 0.019 | Gastric NOS | 0.99 (0.88–1.12) | 0.899 |
| **Year (continuous)** | **0.98 (0.97–0.98)** | **<0.001** | **Year (continuous)** | 0.98 (0.97–0.98) | <0.001 | **Year (continuous)** | 0.98 (0.97–0.98) | <0.001 | **Year (continuous)** | 0.99 (0.99–1.00) | <0.001 |
|  |  |  |  |  |  |  |  |  | **Treatment** |  |  |
|  |  |  |  |  |  |  |  |  | Surgery Yes | 0.51 (0.49–0.54) | <0.001 |
|  |  |  |  |  |  |  |  |  | RT Yes | 1.05 (1.00–1.10) | 0.057 |
|  |  |  |  |  |  |  |  |  | Chemo Yes | 0.48 (0.46–0.50) | <0.001 |

**Table S2B. Full multivariable Cox regression results for overall survival: sensitivity analyses across age specifications and treatment adjustment (n=14,416).** Sensitivity analyses for the final multivariable Cox regression model of overall survival. The Original model used prespecified age strata (<50, 50–70, >70).

**Sensitivity 1** reclassified age as <40, 40–70 and >70.

**Sensitivity 2** modelled age continuously (reported per 10-year increase).

**Sensitivity 3** additionally adjusted the original model for registry-recorded treatment receipt (surgery, radiotherapy [RT], and chemotherapy).

All models adjusted for sex, jurisdiction (North/South), socioeconomic status (SES quintiles plus Unknown), morphology, stage, tumour grade, UGI subsite group, and year of diagnosis (continuous). Hazard ratios (HR) with 95% confidence intervals (CI) are shown; “Ref” indicates the reference category.

| **Co-Variable** | **OS @1y HR (95% CI)** | **CSS @1y HR (95% CI)** | **OS @1y p Value** | **CSS @1y p Value** | **OS @5y HR (95% CI)** | **CSS @5y HR (95% CI)** | **OS @5y p Value** | **CSS @5y p Value** |
| --- | --- | --- | --- | --- | --- | --- | --- | --- |
| **Age group** |  |  |  |  |  |  |  |  |
| <50 | Ref | Ref | Ref | Ref | Ref | Ref | Ref | Ref |
| 50–70 | 1.10 (1.00–1.21) | 1.08 (0.99–1.19) | 0.046 | 0.095 | 1.08 (1.00–1.16) | 1.06 (0.98–1.14) | 0.039 | 0.138 |
| > 70 | 1.87 (1.71–2.05) | 1.80 (1.64–1.98) | <0.001 | <0.001 | 1.73 (1.61–1.86) | 1.60 (1.48–1.72) | <0.001 | <0.001 |
| **Sex** |  |  |  |  |  |  |  |  |
| Female | Ref | Ref | Ref | Ref | Ref | Ref | Ref | Ref |
| Male | 0.94 (0.89–0.99) | 0.92 (0.87–0.97) | 0.014 | 0.002 | 0.95 (0.91–0.99) | 0.92 (0.88–0.96) | 0.016 | <0.001 |
| **Location** |  |  |  |  |  |  |  |  |
| North | Ref | Ref | Ref | Ref | Ref | Ref | Ref | Ref |
| South | 1.05 (0.98–1.11) | 0.93 (0.87–0.99) | 0.145 | 0.018 | 0.98 (0.94–1.03) | 0.80 (0.76–0.84) | 0.516 | <0.001 |
| **SES** |  |  |  |  |  |  |  |  |
| Quintile 1 | Ref | Ref | Ref | Ref | Ref | Ref | Ref | Ref |
| Quintile 2 | 1.08 (1.00–1.17) | 1.07 (0.98–1.17) | 0.065 | 0.131 | 1.04 (0.97–1.11) | 1.05 (0.98–1.12) | 0.275 | 0.204 |
| Quintile 3 | 1.10 (1.01–1.19) | 1.10 (1.02–1.20) | 0.023 | 0.020 | 1.06 (1.00–1.13) | 1.08 (1.01–1.15) | 0.067 | 0.029 |
| Quintile 4 | 1.12 (1.03–1.21) | 1.14 (1.05–1.24) | 0.005 | 0.001 | 1.08 (1.01–1.15) | 1.10 (1.03–1.17) | 0.019 | 0.004 |
| Quintile 5 | 1.03 (0.89–1.19) | 0.98 (0.84–1.15) | 0.720 | 0.810 | 1.12 (1.06–1.19) | 1.14 (1.07–1.21) | <0.001 | <0.001 |
| Unknown | 1.03 (0.89–1.19) | 0.98 (0.84–1.15) | 0.720 | 0.810 | 1.02 (0.91–1.15) | 1.03 (0.90–1.16) | 0.692 | 0.684 |
| **Morphology** |  |  |  |  |  |  |  |  |
| Diffuse | Ref | Ref | Ref | Ref | Ref | Ref | Ref | Ref |
| Intestinal | 0.99 (0.92–1.06) | 0.99 (0.92–1.06) | 0.769 | 0.817 | 0.95 (0.90–1.01) | 0.95 (0.90–1.01) | 0.087 | 0.087 |
| Mucinous | 0.99 (0.84–1.17) | 0.99 (0.83–1.18) | 0.888 | 0.898 | 0.98 (0.86–1.12) | 0.97 (0.84–1.12) | 0.777 | 0.693 |
| Other/Unknown | 0.87 (0.71–1.07) | 0.84 (0.68–1.05) | 0.182 | 0.123 | 0.88 (0.75–1.02) | 0.86 (0.73–1.02) | 0.097 | 0.082 |
| **Stage** |  |  |  |  |  |  |  |  |
| Stage I | Ref | Ref | Ref | Ref | Ref | Ref | Ref | Ref |
| Stage II | 1.78 (1.54–2.05) | 1.88 (1.60–2.21) | <0.001 | <0.001 | 2.00 (1.82–2.19) | 2.38 (2.13–2.65) | <0.001 | <0.001 |
| Stage III | 2.84 (2.50–3.22) | 3.15 (2.73–3.65) | <0.001 | <0.001 | 3.35 (3.08–3.64) | 4.10 (3.71–4.53) | <0.001 | <0.001 |
| Stage IV | 9.24 (8.21–10.40) | 10.36 (9.03–11.87) | <0.001 | <0.001 | 9.29 (8.57–10.06) | 11.22 (10.19–12.36) | <0.001 | <0.001 |
| **Tumour grade** |  |  |  |  |  |  |  |  |
| Grade 1 | Ref | Ref | Ref | Ref | Ref | Ref | Ref | Ref |
| Grade 2 | 1.18 (0.97–1.45) | 1.20 (0.96–1.49) | 0.103 | 0.106 | 1.17 (1.02–1.36) | 1.26 (1.07–1.48) | 0.030 | 0.006 |
| Grade 3 | 1.59 (1.30–1.95) | 1.64 (1.32–2.04) | <0.001 | <0.001 | 1.47 (1.27–1.70) | 1.59 (1.35–1.87) | <0.001 | <0.001 |
| Grade 4 | 1.47 (0.99–2.18) | 1.38 (0.90–2.13) | 0.058 | 0.137 | 1.40 (1.02–1.93) | 1.44 (1.01–2.03) | 0.038 | 0.041 |
| Unknown | 1.54 (1.26–1.89) | 1.51 (1.21–1.88) | <0.001 | <0.001 | 1.44 (1.24–1.67) | 1.44 (1.22–1.71) | <0.001 | <0.001 |
| **UGI subset** |  |  |  |  |  |  |  |  |
| Oesophagus Upper Middle | Ref | Ref | Ref | Ref | Ref | Ref | Ref | Ref |
| Oesophagus Lower Third/OGJ | 0.78 (0.68–0.90) | 0.81 (0.70–0.93) | <0.001 | 0.004 | 0.81 (0.72–0.91) | 0.82 (0.73–0.93) | <0.001 | 0.001 |
| Gastric Cardia | 0.70 (0.61–0.81) | 0.69 (0.60–0.80) | <0.001 | <0.001 | 0.73 (0.65–0.82) | 0.72 (0.63–0.81) | <0.001 | <0.001 |
| Gastric Non Cardia | 0.77 (0.67–0.89) | 0.78 (0.67–0.90) | <0.001 | <0.001 | 0.74 (0.66–0.83) | 0.72 (0.64–0.82) | <0.001 | <0.001 |
| Gastric NOS | 0.92 (0.80–1.06) | 0.92 (0.79–1.07) | 0.247 | 0.265 | 0.88 (0.78–0.99) | 0.85 (0.75–0.96) | 0.030 | 0.012 |
| **Year (continuous)** | 0.97 (0.97–0.98) | 0.98 (0.97–0.98) | <0.001 | <0.001 | 0.98 (0.97–0.98) | 0.98 (0.98–0.98) | <0.001 | <0.001 |

**Table S3.** Multivariable Cox regression comparing overall survival (OS) and cancer-specific survival (CSS) at 1 and 5 years. Models were administratively censored at 12 months (OS@1y, CSS@1y) and 60 months (OS@5y, CSS@5y). CSS used the registry VITAL-CAN cause-of-death indicator (death from index gastro-oesophageal cancer as event; other deaths censored). Hazard ratios (HRs) and 95% confidence intervals (CI) are shown; reference categories are indicated as “Ref”.

| **Cancer** | **Sex** | **Age** | **Timepoint** | **NCRI Net Survival** | **95% Lower** | **95% Upper** | **OS survival South** | **95% lower** | **95% upper** | **OS survival North and South** | **95% lower** | **95% upper** |
| --- | --- | --- | --- | --- | --- | --- | --- | --- | --- | --- | --- | --- |
| OAC | Female | 15-44 | 1 years | **0.55** | 0.23 | 0.78 | **0.55** | 0.23 | 0.78 | **0.53** | 0.26 | 0.74 |
| OAC | Female | 45-54 | 1 years | **0.60** | 0.32 | 0.80 | **0.60** | 0.32 | 0.80 | **0.64** | 0.40 | 0.80 |
| OAC | Female | 55-64 | 1 years | **0.62** | 0.42 | 0.77 | **0.60** | 0.42 | 0.74 | **0.54** | 0.41 | 0.66 |
| OAC | Female | 65-74 | 1 years | **0.62** | 0.48 | 0.74 | **0.58** | 0.45 | 0.68 | **0.57** | 0.47 | 0.66 |
| OAC | Female | 75-99 | 1 years | **0.51** | 0.38 | 0.62 | **0.43** | 0.33 | 0.52 | **0.40** | 0.32 | 0.48 |
| OAC | Male | 15-44 | 1 years | **0.50** | 0.27 | 0.69 | **0.50** | 0.27 | 0.69 | **0.56** | 0.35 | 0.72 |
| OAC | Male | 45-54 | 1 years | **0.64** | 0.55 | 0.72 | **0.64** | 0.54 | 0.72 | **0.65** | 0.57 | 0.71 |
| OAC | Male | 55-64 | 1 years | **0.65** | 0.58 | 0.71 | **0.64** | 0.57 | 0.70 | **0.64** | 0.59 | 0.68 |
| OAC | Male | 65-74 | 1 years | **0.60** | 0.53 | 0.66 | **0.59** | 0.53 | 0.65 | **0.56** | 0.51 | 0.60 |
| OAC | Male | 75-99 | 1 years | **0.41** | 0.34 | 0.48 | **0.38** | 0.32 | 0.44 | **0.42** | 0.37 | 0.47 |
| OAC | Female | 15-44 | 5 years | **0.18** | 0.04 | 0.41 | **0.18** | 0.03 | 0.44 | **0.27** | 0.08 | 0.50 |
| OAC | Female | 45-54 | 5 years | **0.20** | 0.05 | 0.42 | **0.20** | 0.05 | 0.42 | **0.23** | 0.08 | 0.41 |
| OAC | Female | 55-64 | 5 years | **0.46** | 0.27 | 0.63 | **0.37** | 0.22 | 0.53 | **0.36** | 0.24 | 0.48 |
| OAC | Female | 65-74 | 5 years | **0.31** | 0.19 | 0.45 | **0.30** | 0.20 | 0.41 | **0.28** | 0.19 | 0.37 |
| OAC | Female | 75-99 | 5 years | **0.07** | 0.02 | 0.15 | **0.06** | 0.03 | 0.12 | **0.07** | 0.04 | 0.12 |
| OAC | Male | 15-44 | 5 years | **0.14** | 0.03 | 0.33 | **0.13** | 0.03 | 0.33 | **0.10** | 0.02 | 0.25 |
| OAC | Male | 45-54 | 5 years | **0.27** | 0.19 | 0.35 | **0.26** | 0.19 | 0.34 | **0.28** | 0.21 | 0.34 |
| OAC | Male | 55-64 | 5 years | **0.33** | 0.26 | 0.39 | **0.31** | 0.25 | 0.37 | **0.31** | 0.26 | 0.36 |
| OAC | Male | 65-74 | 5 years | **0.26** | 0.20 | 0.32 | **0.24** | 0.19 | 0.29 | **0.21** | 0.17 | 0.25 |
| OAC | Male | 75-99 | 5 years | **0.09** | 0.05 | 0.15 | **0.06** | 0.03 | 0.09 | **0.09** | 0.06 | 0.12 |
| GC | Female | 15-44 | 1 years | **0.71** | 0.57 | 0.82 | **0.56** | 0.38 | 0.71 | **0.49** | 0.35 | 0.62 |
| GC | Female | 45-54 | 1 years | **0.73** | 0.63 | 0.81 | **0.62** | 0.49 | 0.72 | **0.58** | 0.47 | 0.68 |
| GC | Female | 55-64 | 1 years | **0.63** | 0.53 | 0.71 | **0.56** | 0.46 | 0.65 | **0.56** | 0.47 | 0.64 |
| GC | Female | 65-74 | 1 years | **0.64** | 0.58 | 0.70 | **0.60** | 0.53 | 0.66 | **0.57** | 0.51 | 0.63 |
| GC | Female | 75-99 | 1 years | **0.39** | 0.34 | 0.45 | **0.36** | 0.31 | 0.41 | **0.34** | 0.30 | 0.38 |
| GC | Male | 15-44 | 1 years | **0.62** | 0.50 | 0.72 | **0.50** | 0.36 | 0.62 | **0.47** | 0.35 | 0.58 |
| GC | Male | 45-54 | 1 years | **0.68** | 0.60 | 0.75 | **0.64** | 0.55 | 0.72 | **0.60** | 0.52 | 0.67 |
| GC | Male | 55-64 | 1 years | **0.68** | 0.62 | 0.72 | **0.65** | 0.59 | 0.70 | **0.62** | 0.57 | 0.67 |
| GC | Male | 65-74 | 1 years | **0.61** | 0.56 | 0.66 | **0.58** | 0.53 | 0.62 | **0.55** | 0.51 | 0.59 |
| GC | Male | 75-99 | 1 years | **0.42** | 0.38 | 0.47 | **0.42** | 0.38 | 0.46 | **0.43** | 0.40 | 0.46 |
| GC | Female | 15-44 | 5 years | **0.48** | 0.34 | 0.61 | **0.21** | 0.09 | 0.35 | **0.22** | 0.12 | 0.35 |
| GC | Female | 45-54 | 5 years | **0.57** | 0.46 | 0.67 | **0.38** | 0.27 | 0.50 | **0.38** | 0.28 | 0.48 |
| GC | Female | 55-64 | 5 years | **0.41** | 0.32 | 0.50 | **0.23** | 0.15 | 0.32 | **0.23** | 0.17 | 0.31 |
| GC | Female | 65-74 | 5 years | **0.40** | 0.34 | 0.47 | **0.27** | 0.22 | 0.34 | **0.25** | 0.20 | 0.30 |
| GC | Female | 75-99 | 5 years | **0.16** | 0.12 | 0.21 | **0.09** | 0.06 | 0.13 | **0.09** | 0.07 | 0.12 |
| GC | Male | 15-44 | 5 years | **0.38** | 0.27 | 0.49 | **0.18** | 0.09 | 0.30 | **0.15** | 0.08 | 0.24 |
| GC | Male | 45-54 | 5 years | **0.33** | 0.25 | 0.41 | **0.23** | 0.16 | 0.31 | **0.22** | 0.16 | 0.28 |
| GC | Male | 55-64 | 5 years | **0.33** | 0.28 | 0.38 | **0.26** | 0.21 | 0.31 | **0.24** | 0.20 | 0.29 |
| GC | Male | 65-74 | 5 years | **0.34** | 0.29 | 0.39 | **0.27** | 0.23 | 0.31 | **0.24** | 0.21 | 0.28 |
| GC | Male | 75-99 | 5 years | **0.19** | 0.15 | 0.23 | **0.13** | 0.10 | 0.16 | **0.13** | 0.11 | 0.15 |

**Table S4. Net survival and overall survival comparison**. NCRI net survival and observed overall survival (OS) at 1 and 5 years by cancer site, sex and age group, 2014–2018. The 2014–2018 period was selected to allow mature 5-year follow-up. Net survival estimates were obtained from the National Cancer Registry Ireland (NCRI) and represent survival adjusted for background (non-cancer) mortality. Observed OS estimates were calculated from the all-island cohort for (i) Republic of Ireland only (“South”) and (ii) the pooled all-island cohort (“North+South”). Values are proportions with 95% confidence intervals (CI). OAC = oesophageal adenocarcinoma; GC = gastric adenocarcinoma.

***
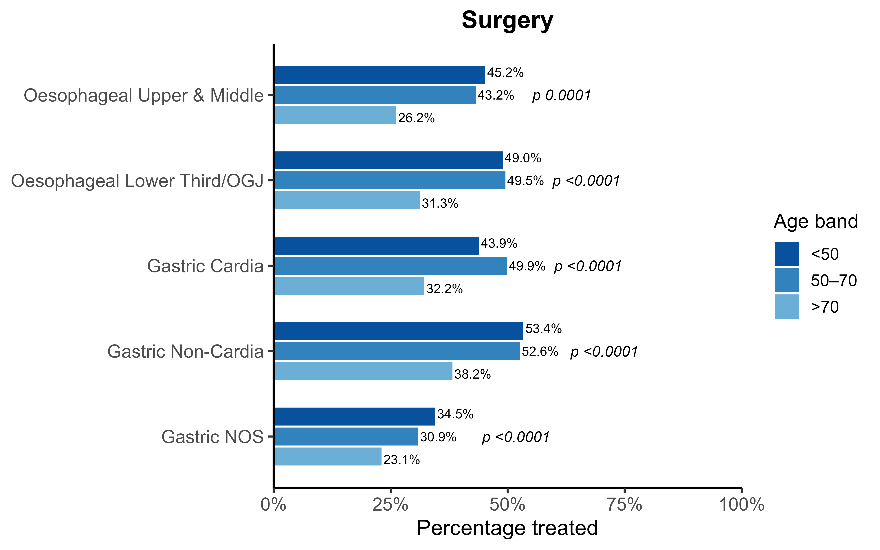

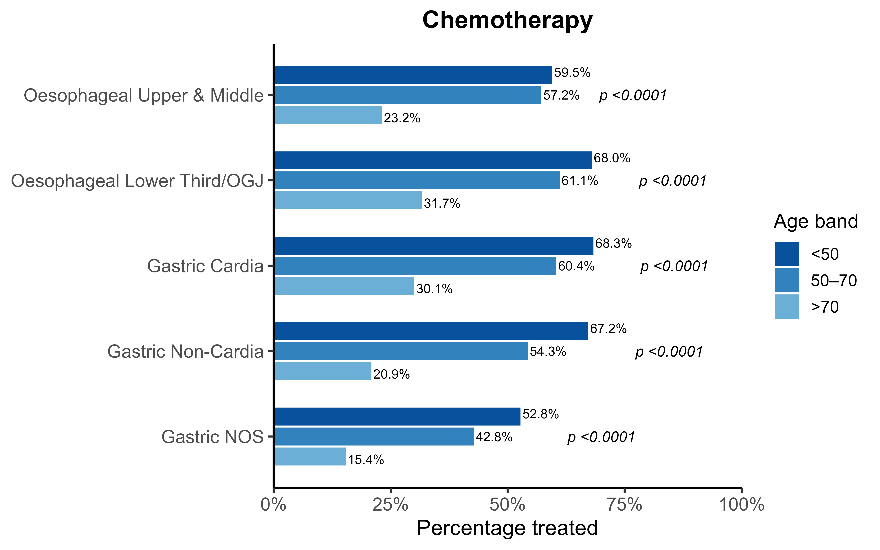

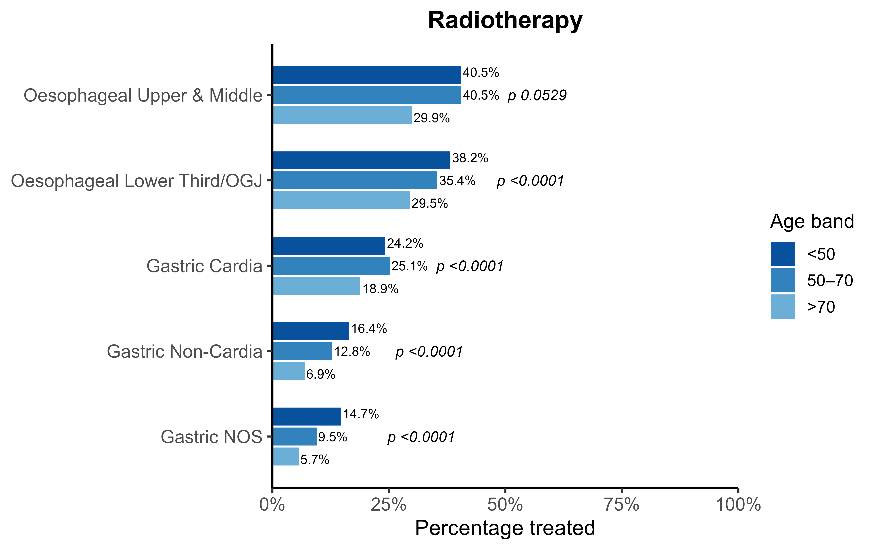

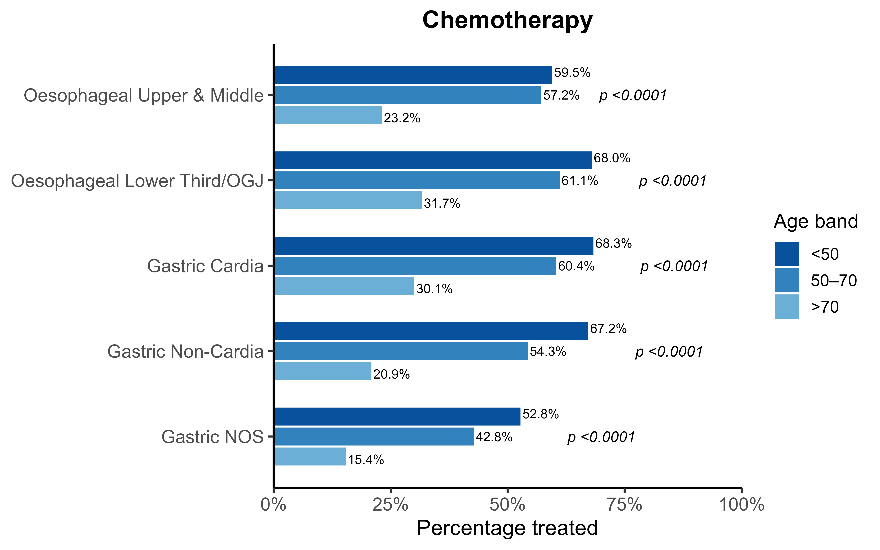

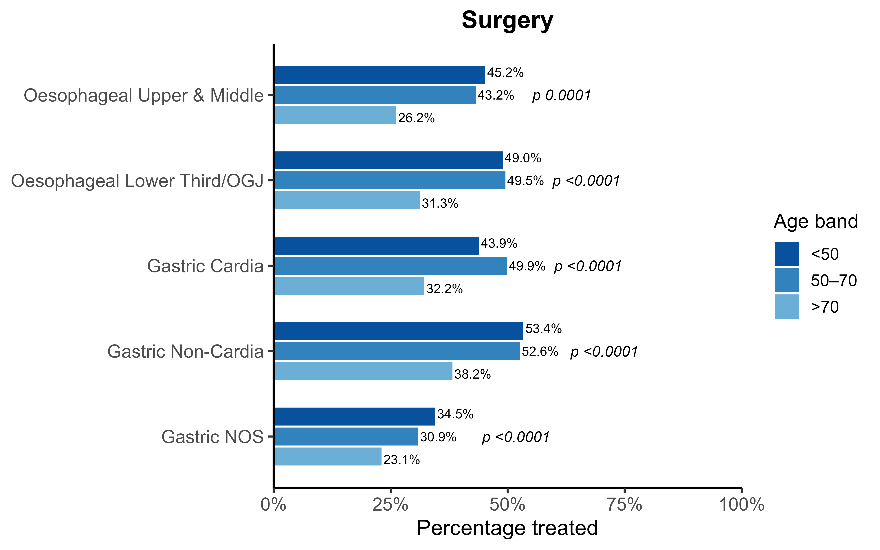
***

**B**

**A**

**A**

**B**

**C**

**Figure S7 Treatment uptake by anatomical subsite and age group.** Treatment subtypes include surgery (A), chemotherapy (B) and radiotherapy (C). Bars represent the percentage of patients in each age band who received the indicated therapy, stratified by tumour site (Oesophageal Upper & Middle; Oesophageal Lower Third/OGJ; Gastric Cardia; Gastric Non-Cardia; Gastric NOS). P-values to the right of each site’s bar cluster derive from Pearson’s χ² test comparing treatment rates across the three age bands within that subsite (p < 0.05 indicates a significant age effect)., OGJ = Oesophagogastric junction, NOS = not otherwise specified
