# Supplementary material for: Real-world insights into young-onset gastroesophageal adenocarcinoma: an all-Ireland population-based cancer registry analysis
Source: ESMO Gastrointest Oncol. 2026 Apr 17;12:100325. doi: 10.1016/j.esmogo.2026.100325 (PMC13098464; doi:10.1016/j.esmogo.2026.100325)
Supplement: Supplementary Figs [file mmc2.docx]

Supplemental Material


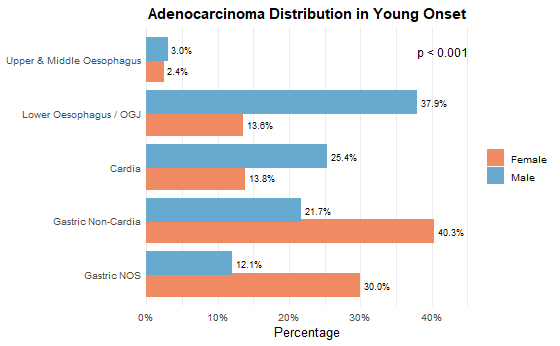


**A**


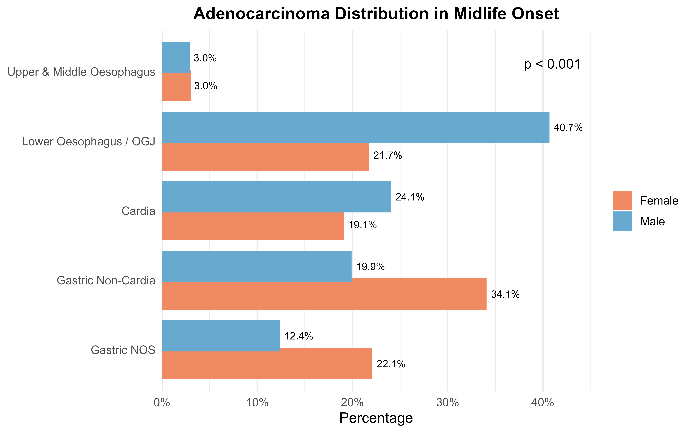


**B**


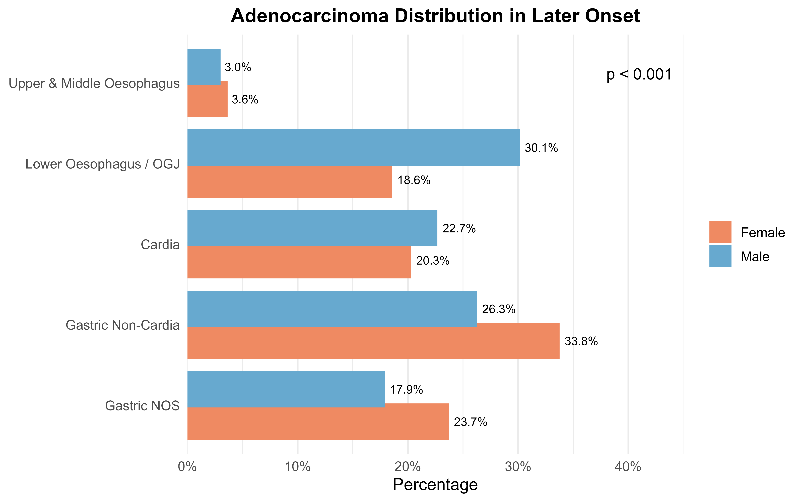


**C**

**Figure S1. Site-specific distribution of adenocarcinoma by sex within each age group:** (A) young-onset (YO, < 50 y), (B) midlife onset (MO, 50–70 y) and (C) later-onset (LO, > 70 y). Bars show the proportion of male (blue) and female (orange) cases for each anatomical sub-site. Across all age bands, men are more likely to present with proximal tumours—the lower oesophagus/OGJ and gastric cardia—whereas women predominate in distal, non-cardia gastric disease. The global Pearson χ² p-value tests whether the male-to-female distribution differs significantly across sub-sites within the displayed age band.

**A**

**B**

**C**

**D**


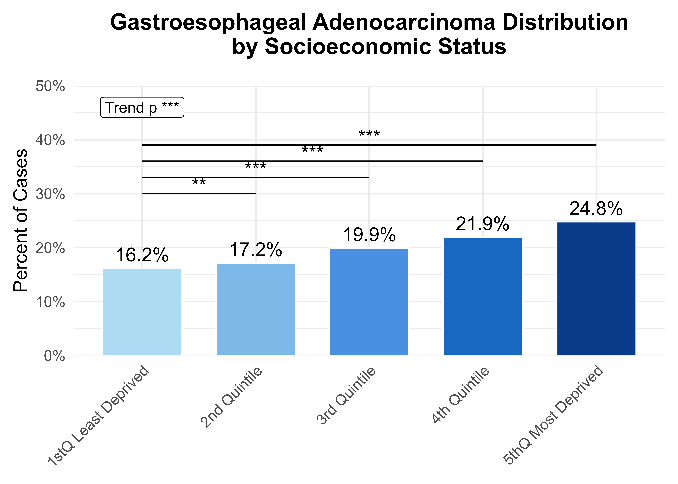


***
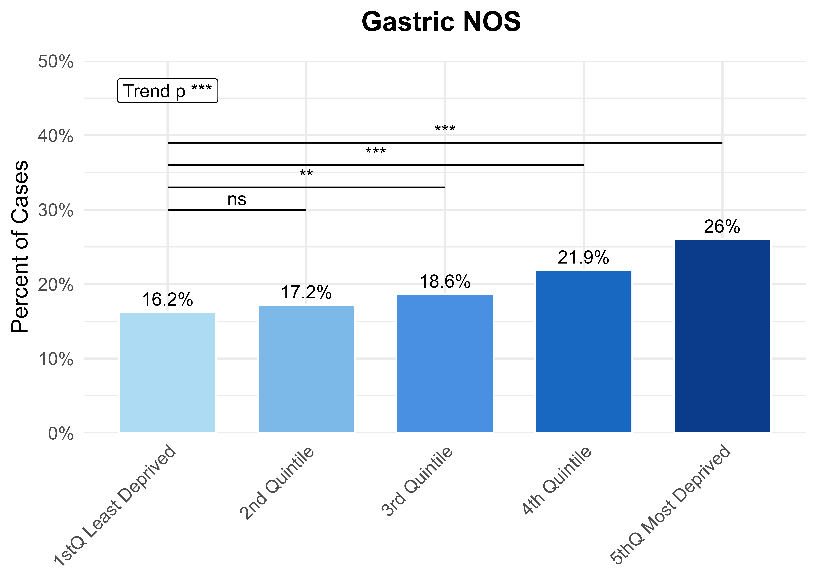

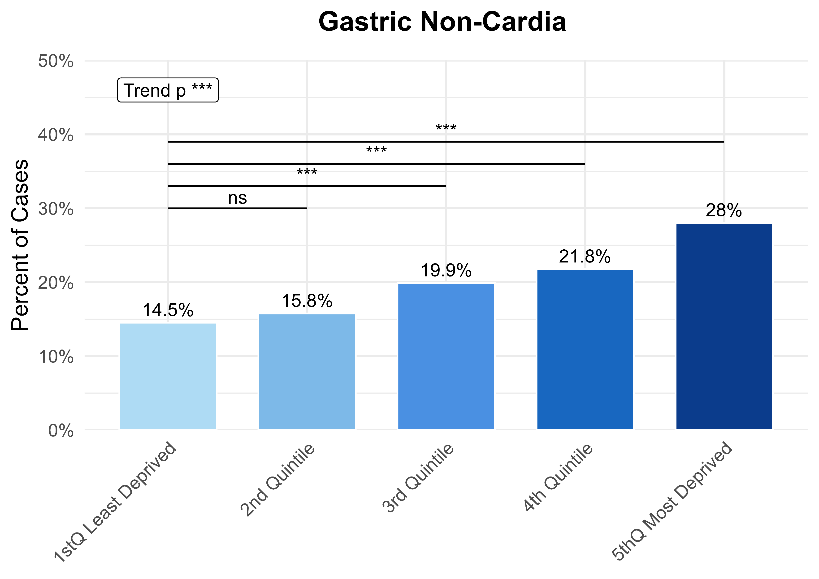

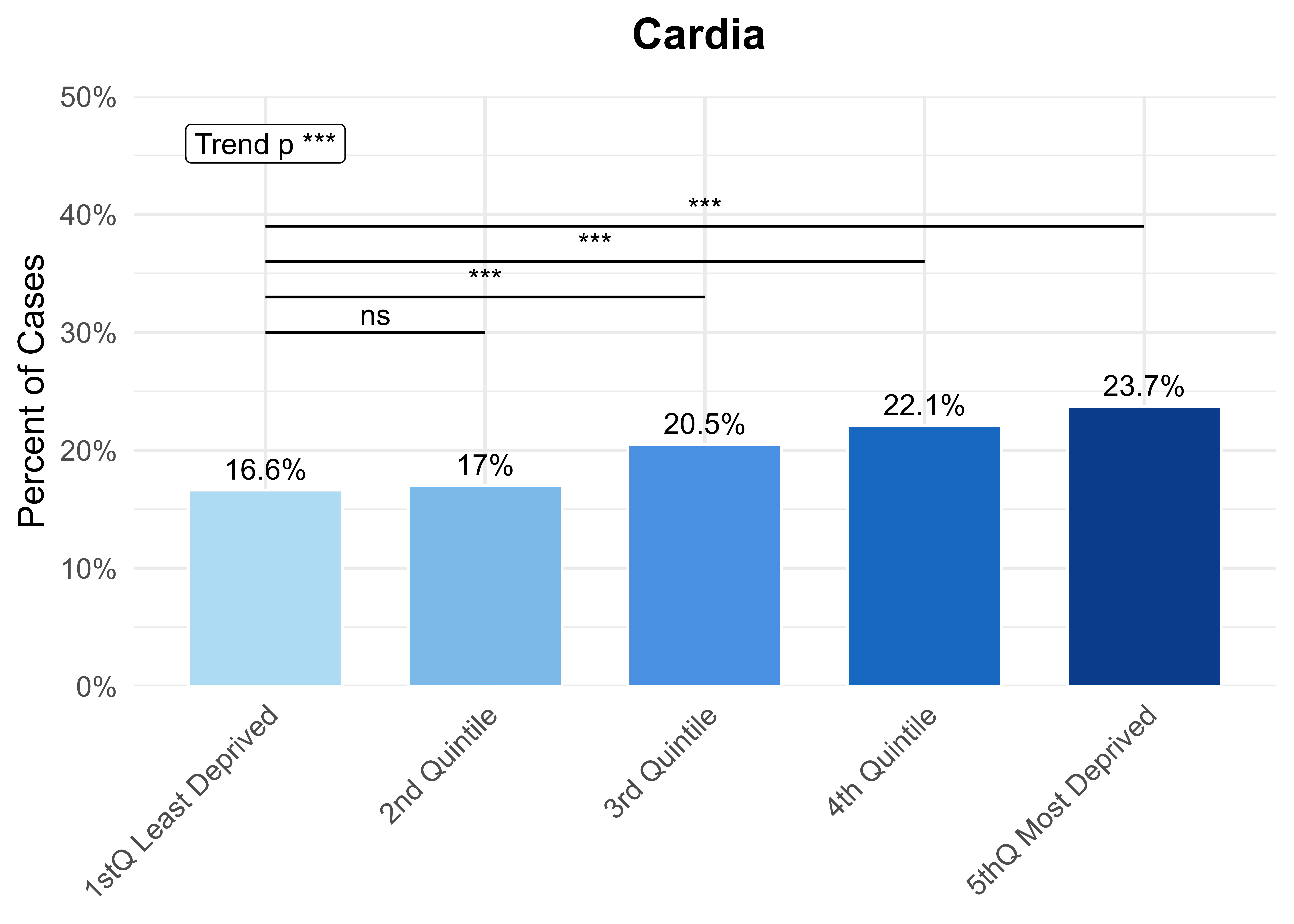
***
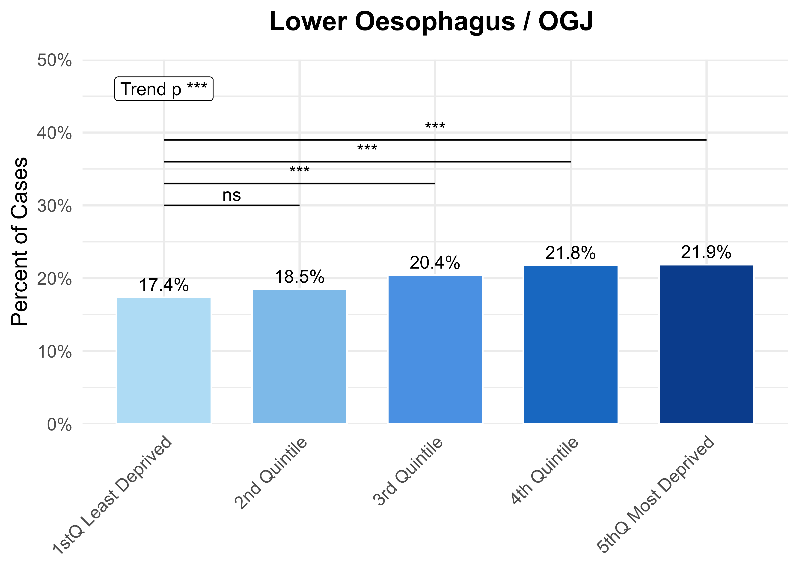


**E**


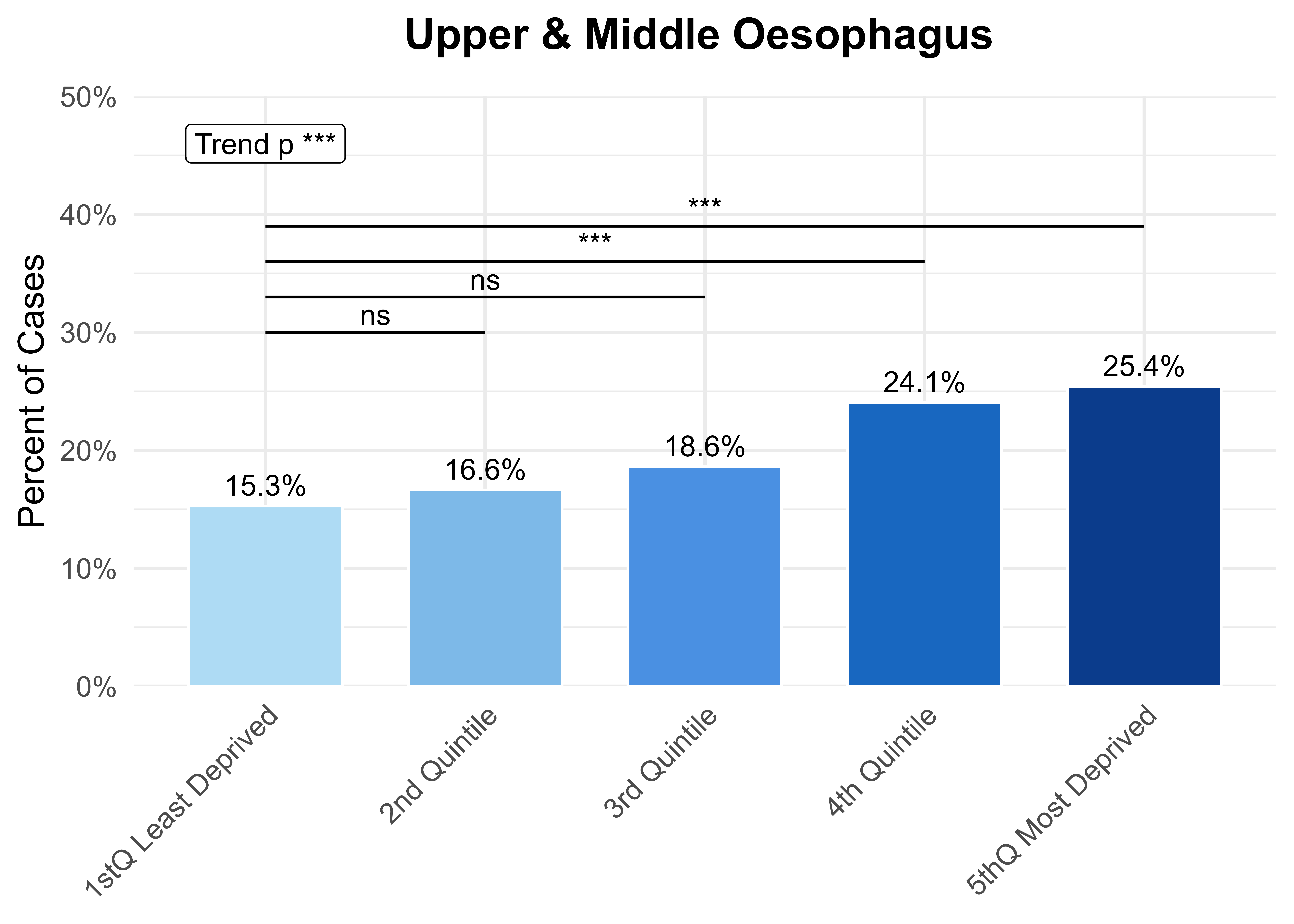


**F**

**Figure S2** Distribution of gastroesophageal adenocarcinoma cases by deprivation quintile and UGI Cancer cases overall (A) and by tumour sites (B-F). A Cochran–Armitage trend test reveals a significant, stepwise increase in case proportion with greater deprivation overall and across tumour types (trend *p* < 0.001). Pairwise comparisons between the least-deprived reference group and each higher quintile are indicated by asterisks: ****** *p* < 0.01, ******* *p* < 0.001. NS= not significant, UGI= Upper Gastrointestinal, OGJ = Oesophagogastric junction, NOS = not otherwise specified.


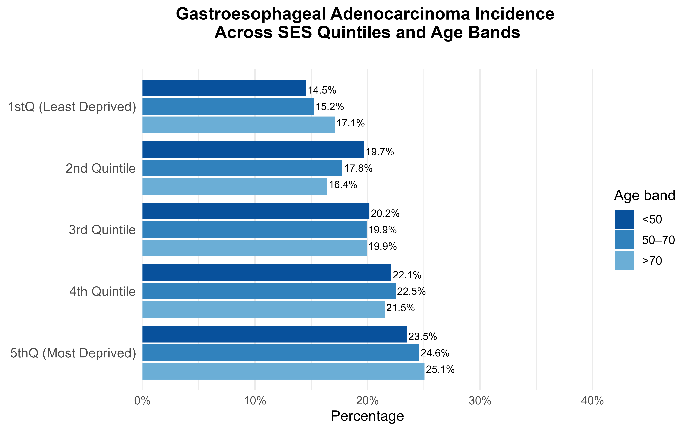


**A**

**B**

| SES Quantile | <50 | 50-70 | >70 |
| --- | --- | --- | --- |
| Q1 vs Q2 p value | 0.000300605 | 1.1702E-05 | 0.179086 |
| Q1 vs Q3 p value | 8.17181E-05 | 3.50083E-15 | 2.73E-08 |
| Q1 vs Q4 p value | 1.93363E-07 | 1.95884E-32 | 1.48E-17 |
| Trend p value | 1.00629E-09 | 4.49308E-63 | 2.52E-71 |

**Figure S3** Distribution of gastroesophageal adenocarcinoma cases by deprivation quintiles with age A) Bar chart showing the percentage of cases across socioeconomic quintile, stratified by age group. A clear, stepwise increase in incidence is observed with increasing deprivation, consistent across all age bands.

(B) Table summarizing pairwise and trend analyses p values. Pairwise comparisons between the least-deprived reference group (Q1) and each higher quintile were performed using chi-square tests, while trend p-values were derived from the Cochran–Armitage test to evaluate linear gradients across quintiles. All age groups demonstrated significant deprivation gradients.


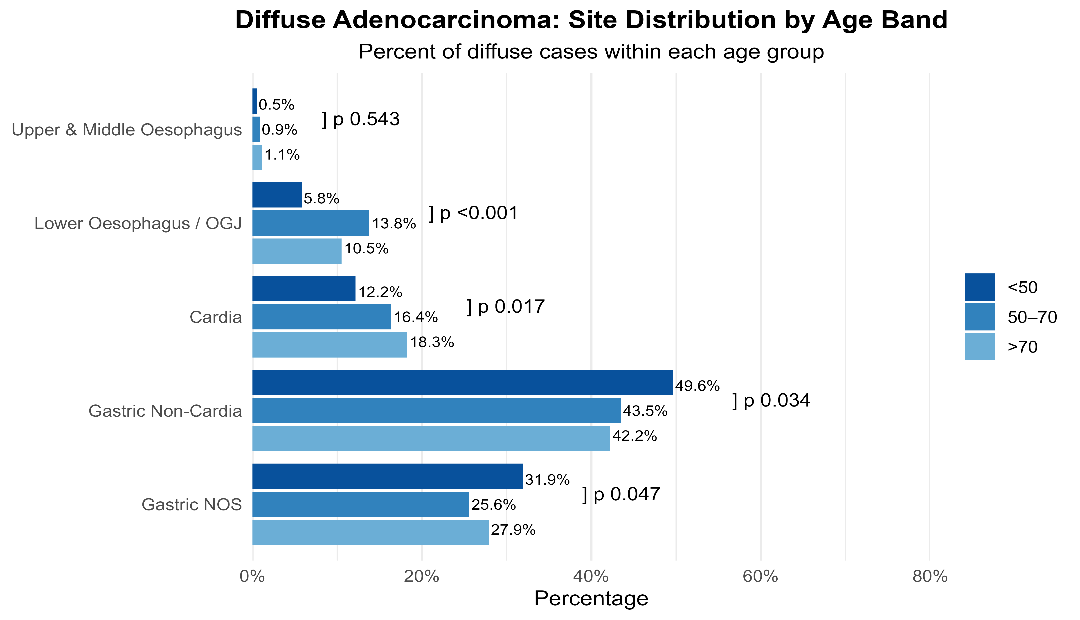


**A**


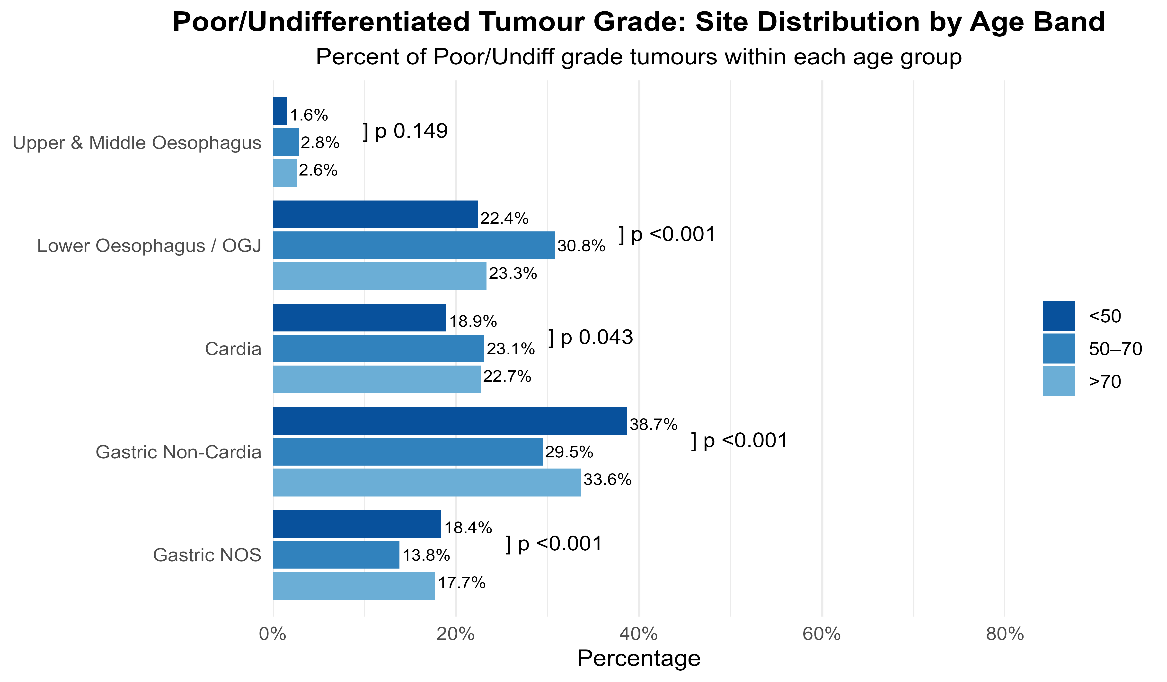


**B**

**Figure S4. Distribution of tumour morphology and grade across anatomical sites by age. Panel A:** Diffuse-type adenocarcinomas are concentrated in the lower oesophagus/OGJ (13.8%) and gastric non-cardia (49.6%) in young-onset cases, shifting toward cardia and gastric NOS in older groups**. Panel B:** Poorly/undifferentiated tumours show a similar pattern, with enrichment in lower oesophagus/OGJ (22.4%) and non-cardia (38.7%) among younger patients. Site-specific distributions differ significantly across age bands (Pearson χ² p-values shown). NOS = not otherwise specified**.**


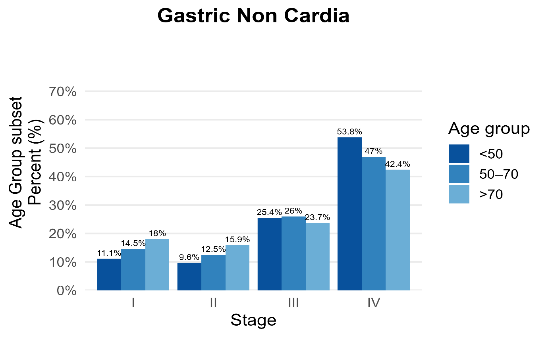

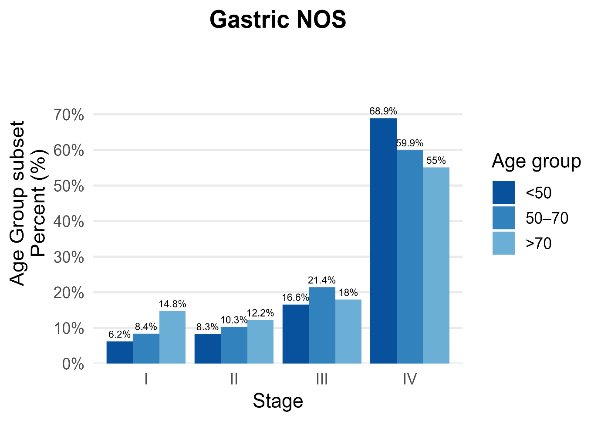

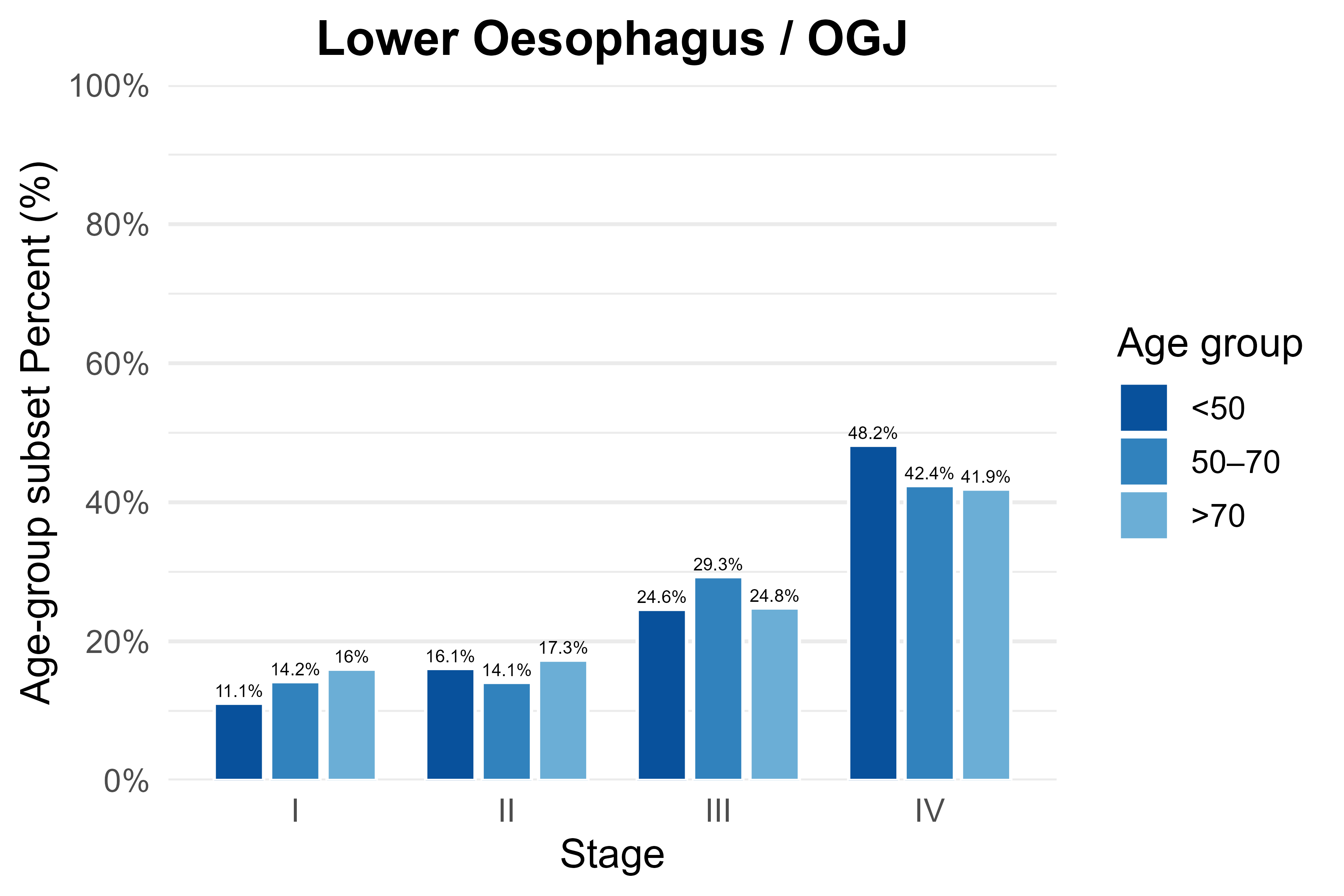

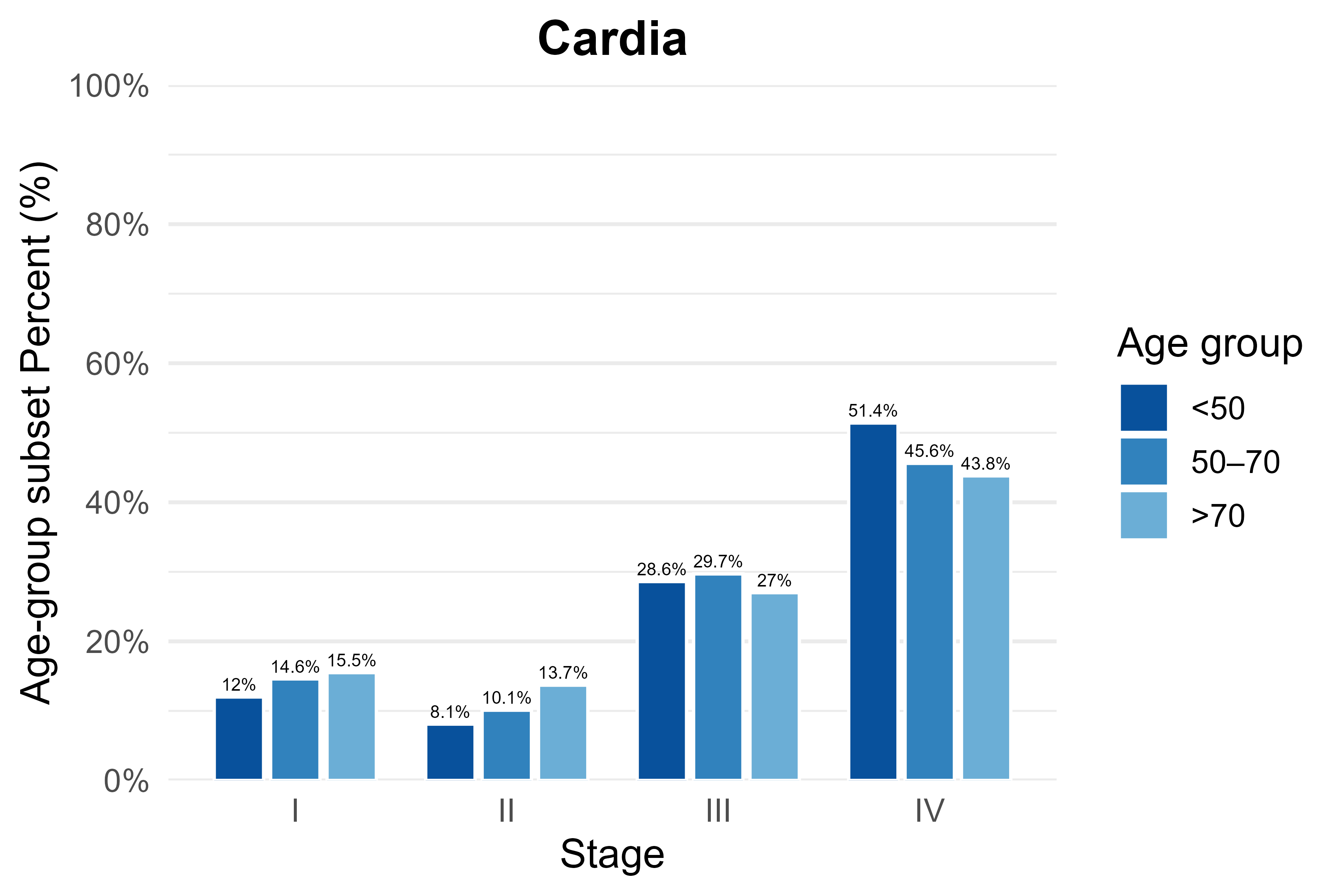


**A**

**B**

**C**

**D**

**E**

| Site | Chi2 p | p YO vs AO | p YO vs LO | Trend p |
| --- | --- | --- | --- | --- |
| Lower Oesophagus / OGJ | 0.099 | 0.025 | 0.020 | 0.127 |
| Cardia | 0.061 | 0.047 | 0.012 | 0.031 |
| Gastric Non-Cardia | <0.001 | 0.013 | <0.001 | <0.001 |
| Gastric NOS | <0.001 | 0.013 | <0.001 | <0.001 |

**Figure S5 Stage at diagnosis by tumour site and age group.** Stacked bar charts display the proportion of cases in each stage (I–IV) within the three age bands—for four anatomical subsites (A-D). Inset Table (E): Statistical comparisons of Stage IV versus earlier-stage disease across age groups by anatomical subsite. Reported p-values include:

(i) Chi² p – a 3 × 2 chi-square test comparing the proportion of Stage IV cases across the three age groups (< 50, 50–70, > 70 years);

(ii) YO vs AO/LO p – one-sided Fisher’s exact tests restricted to Stage IV disease, comparing young-onset (YO) with midlife- (MO) and later-onset (LO) cohorts;

(iii) Trend p – a Cochran–Armitage test assessing a linear trend in the proportion of Stage IV disease across ascending age bands (< 50 → 50–70 → > 70).

Abbreviations: UGI = Upper Gastrointestinal; OGJ = Oesophagogastric junction; NOS = Not Otherwise Specified.


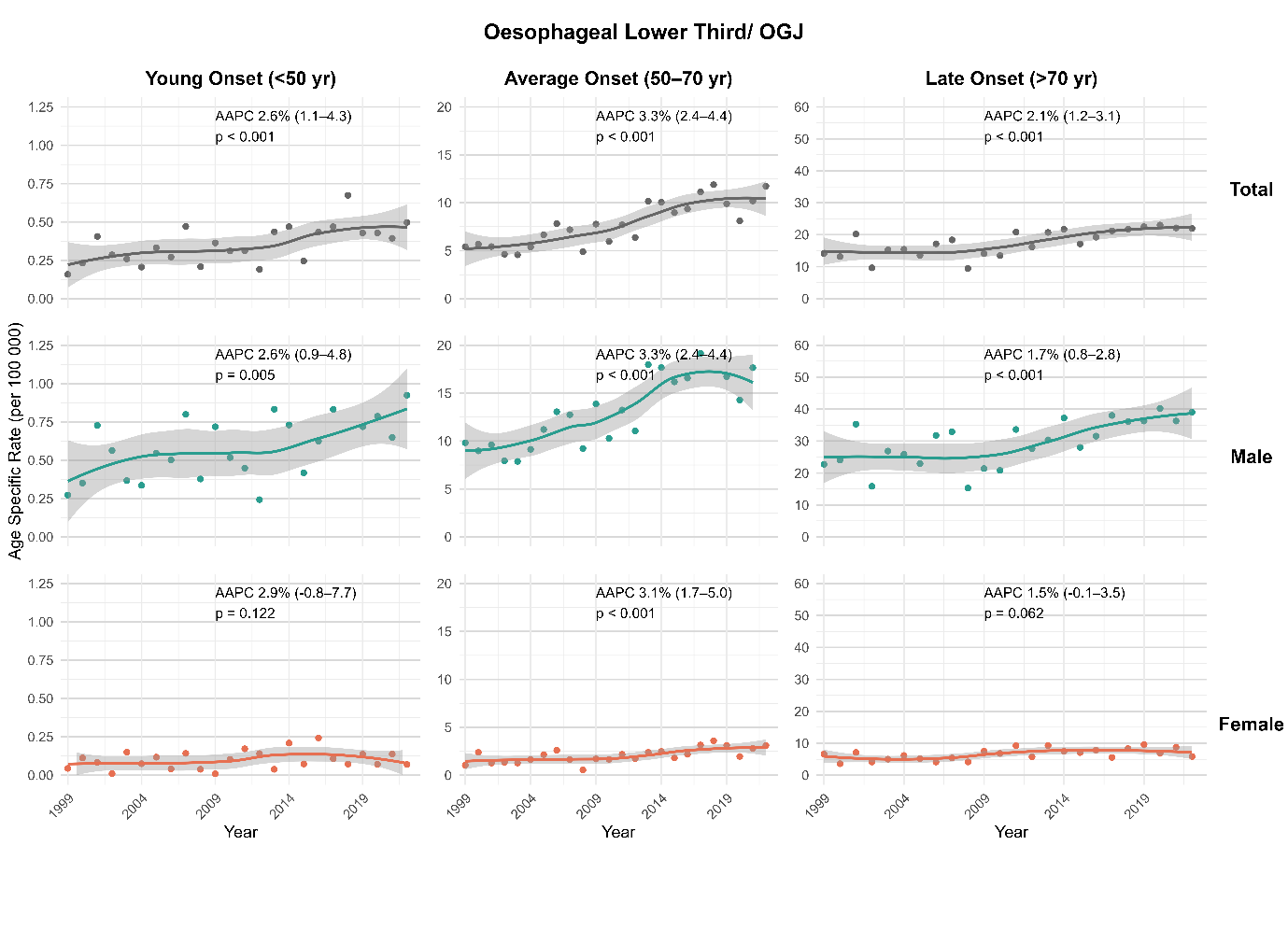


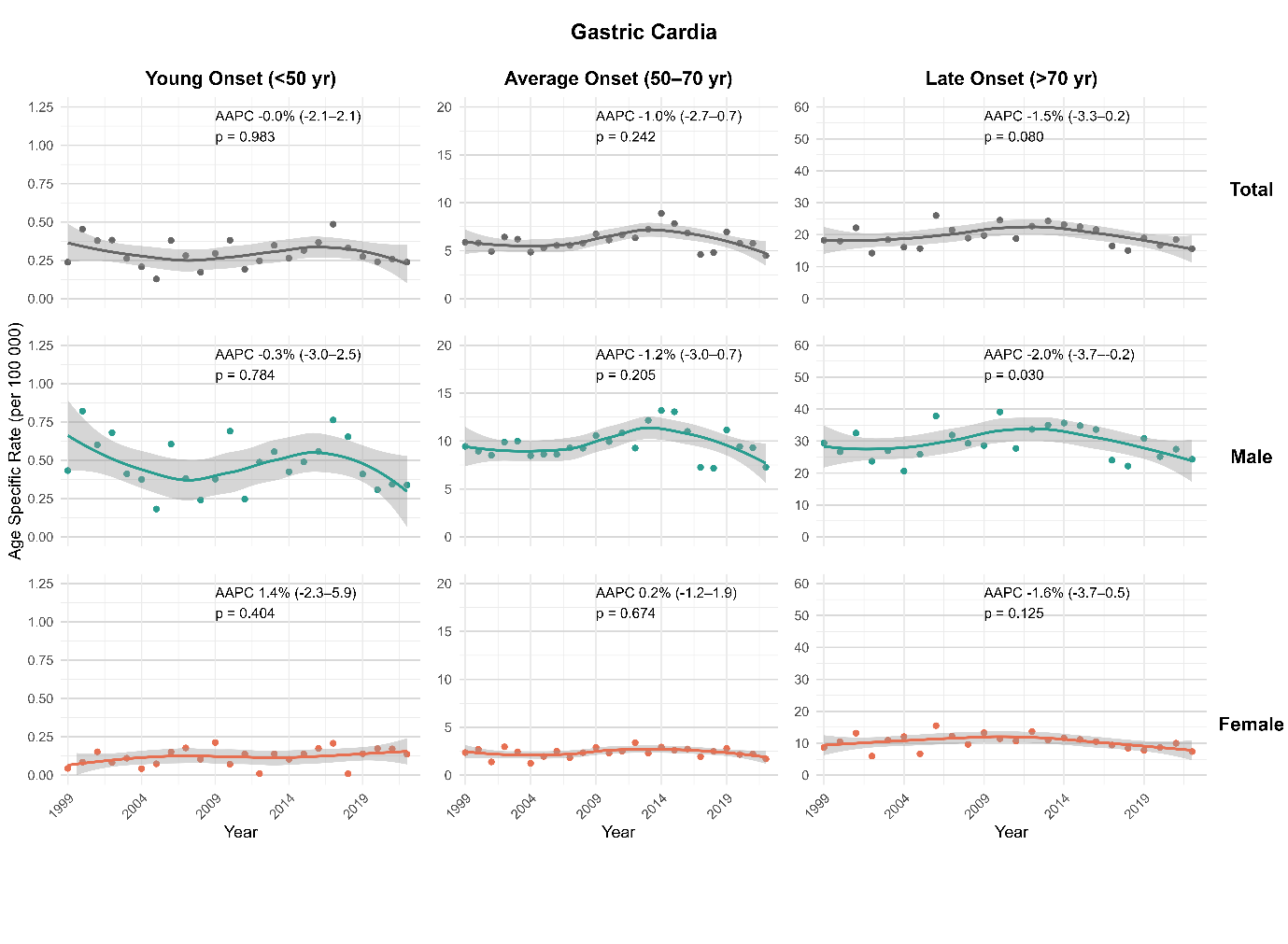


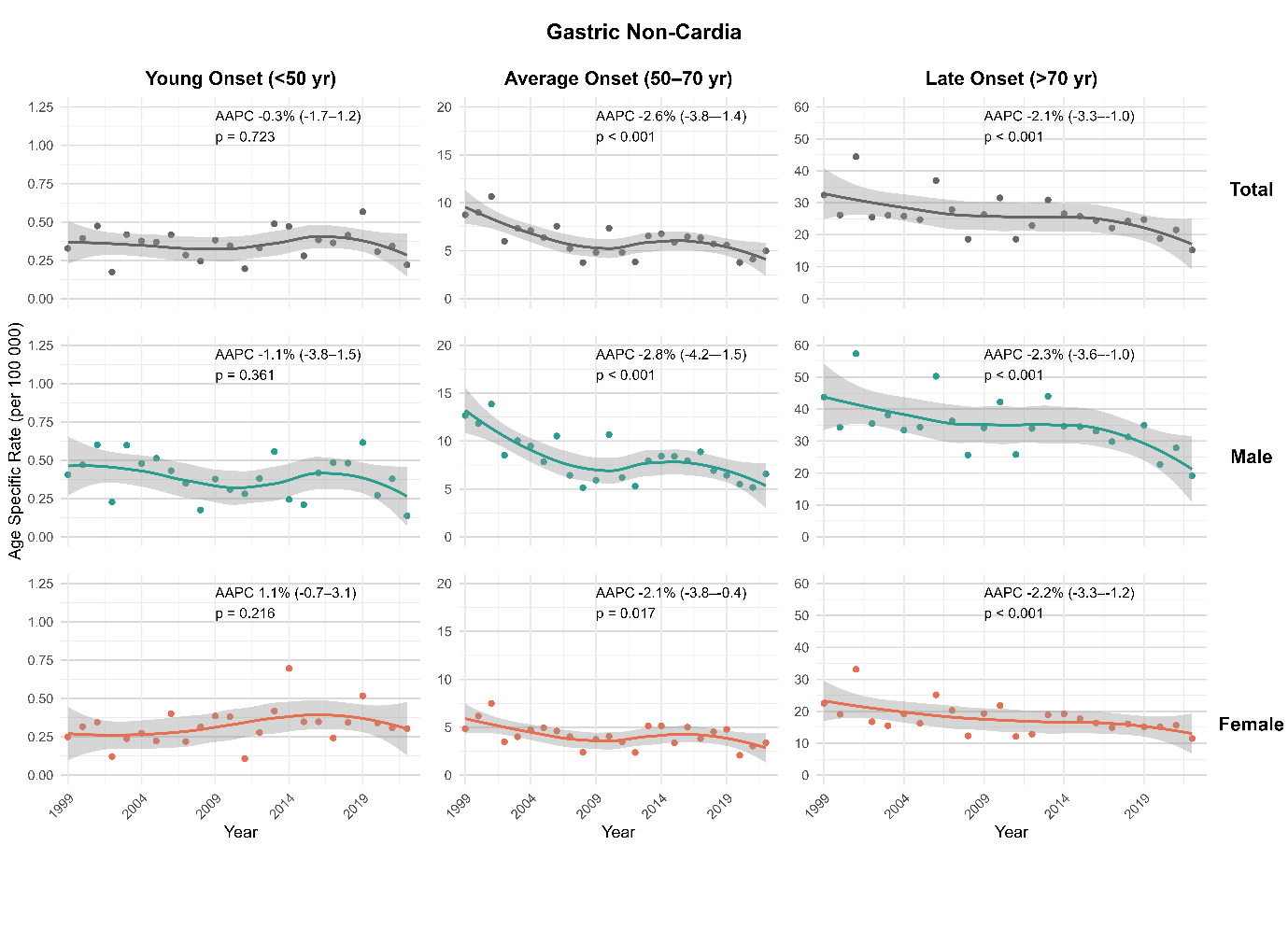


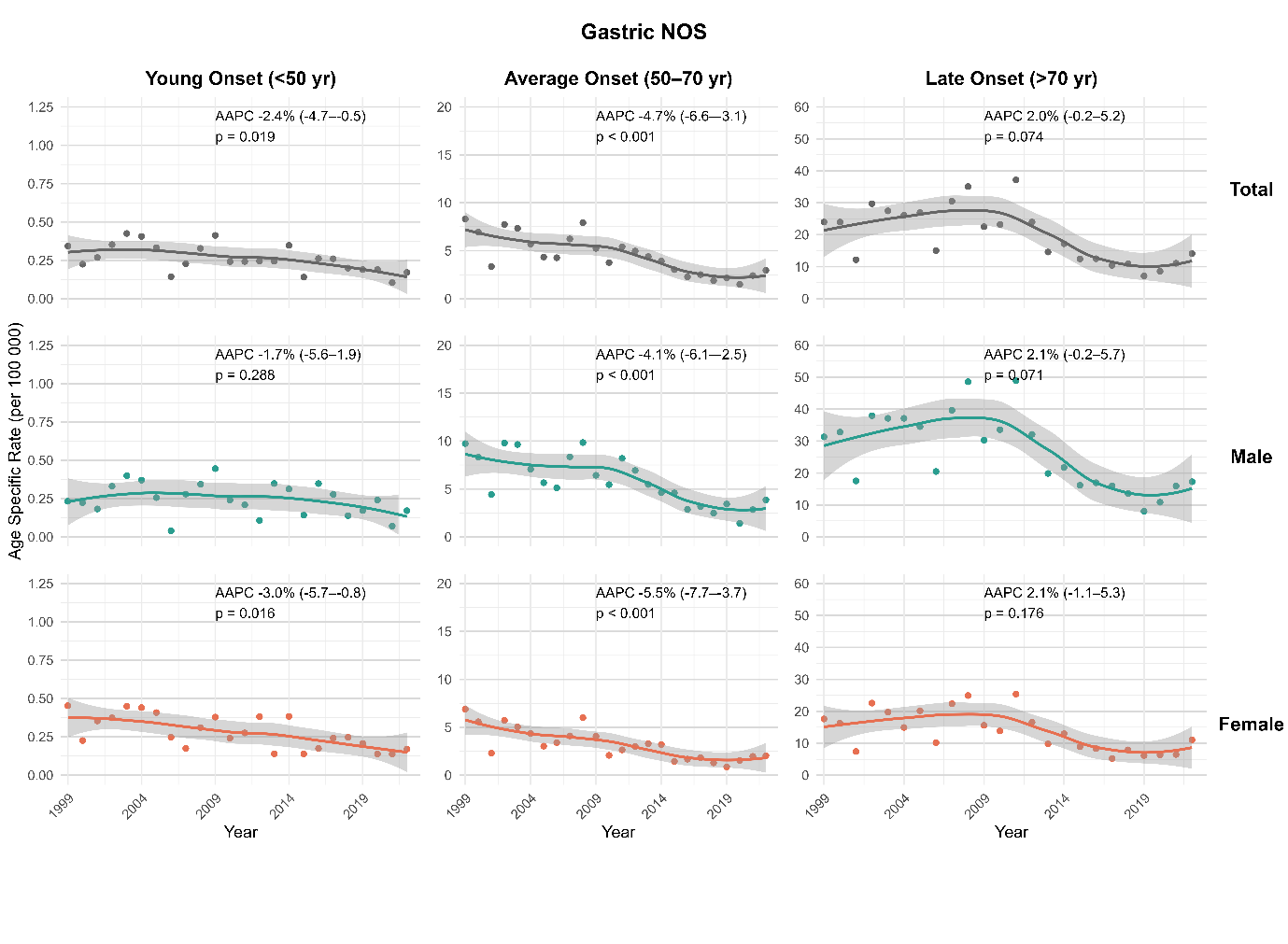


**Figure S6 Age Specific Rate per 100,000 by sub-site, age band and sex**. Annual rates (points) are smoothed with LOESS (Locally Estimated Scatterplot Smoothing) and 95 % CIs (shaded). Panels report APC or AAPC from JoinPoint with 95 % CI and *p*. (A) Lower oesophagus/OGJ (B) Oesophageal NOS, (C) Gastric cardia, (D) Gastric non-cardia, (E) Gastric NOS, OGJ=Oesophagogastric junction. NOS= Not otherwise specified, APC = Annual Percent Change, AAPC = Average APC, CI= Confidence Intervals. P values generated by JoinPoint Regression Analysis software.

| **Variable** | **Alive n(%)** | **Dead n(%)** | **Median Survival months**  **5^th^, 95^th^ CI** | **P Value** |
| --- | --- | --- | --- | --- |
| **Sex** |  |  |  | p = 0.130 |
| - **Males** | 2980 (15.2%) | 16580 (84.8%) | 9.4 (0.6–131.3) |  |
| - **Females** | 1746 (16.1%) | 9091 (83.9%) | 8.2 (0.4–140.7) |  |
| **Age** |  |  |  | p <0.001 |
| - **<50** | 537 (27.4%) | 1425 (72.6%) | 14.7 (1.0–195.5) |  |
| - **50-70** | 2460 (21.2%) | 9139 (78.8%) | 13.0 (0.8–163.6) |  |
| - **>70** | 1729 (10.3%) | 15107 (89.7%) | 6.4 (0.4–90.9) |  |
| **Socioeconomic Status** |  |  |  | P = 0.307 |
| - **1^st^ Q** | 293 (13.2%) | 1931 (86.8%) | 7.3 (0.4–127.8) |  |
| - **2^nd^ Q** | 298 (13.6%) | 1900 (86.4%) | 7.8 (0.4–123.8) |  |
| - **3^rd^ Q** | 262 (13.8%) | 1636 (86.2%) | 8.2 (0.4–118.6) |  |
| - **4^th^ Q** | 265 (14.4%) | 1574 (85.6%) | 8.4 (0.5–130.4) |  |
| - **5^th^ Q** | 217 (13.0%) | 1450 (87.0%) | 8.0 (0.4–118.6) |  |
| **UGI Subset** |  |  |  | p <0.001 |
| - **Oesophageal Upper and Middle** | 97 (15.0%) | 548 (85.0%) | 10.0 (0.8–124.6) |  |
| - **Oesophageal Lower 3^rd^/ OGJ** | 1131 (18.0%) | 5139 (82.0%) | 11.1 (0.9–124.1) |  |
| - **Gastric Cardia** | 725 (15.6%) | 3928 (84.4%) | 11.4 (0.8–127.3) |  |
| - **Gastric Non Cardia** | 845 (15.1%) | 4733 (84.9%) | 9.7 (0.6–145.2) |  |
| - **Gastric NOS** | 293 (8.0%) | 3369 (92.0%) | 5.8 (0.4–123.7) |  |
| **Stage** |  |  |  | p <0.001 |
| - **Stage I** | 1502 (51.4%) | 1419 (48.6%) | 49.6 (2.0–194.9) |  |
| - **Stage II** | 824 (28.5%) | 2069 (71.5%) | 24.6 (2.1–175.4) |  |
| - **Stage III** | 896 (18.2%) | 4040 (81.8%) | 14.4 (1.4–130.7) |  |
| - **Stage IV** | 422 (4.7%) | 8540 (95.3%) | 4.5 (0.4–29.8) |  |

**Table S1 Overall survival by demographic and clinicopathological factors.** Outcome (alive and dead) column percentages refer to the proportion of the total cohort in each category who were alive or deceased at censoring time, December 2022. Median OS is presented with the 5ᵗʰ–95ᵗʰ percentile confidence interval. P-values are from global log-rank tests comparing Kaplan–Meier survival curves across the levels of each variable. SES quintiles (1 = least deprived; 5 = most deprived) derive from the 2019 Index of Multiple Deprivation for cases diagnosed in the Northa and Pobal Index for cases derived in the South. Abbreviations: OGJ = oesophagogastric junction; NOS = not otherwise specified; OS = overall survival; CI = confidence interval.
